# Scalable manufacturing of human iPSC-derived functional liver organoids for acute liver failure treatment

**DOI:** 10.7150/thno.137714

**Published:** 2026-09-18

**Authors:** Xiaoshan Deng, Yunzhong Nie, Yoshihito Hayashi, Na Luo, Yang Li, Riana Plummer, Qinglin Li, Toshiharu Kasai, Takashi Okumura, Yu Kamishibahara, Takemasa Komoto, Takuya Ohkuma, Yasuhiro Yamada, Yumiko Isobe, Kiyoshi Yamaguchi, Satoshi Okamoto, Yasuharu Ueno, Naoki Tanimizu, Yoichi Furukawa, Hideki Taniguchi

**Affiliations:** 1 Division of Regenerative Medicine, Center for Stem Cell Biology and Regenerative Medicine, The Institute of Medical Science, The University of Tokyo, Minato, Tokyo, Japan.; 2 Department of Computational Biology and Medical Sciences, Graduate School of Frontier Sciences, The University of Tokyo, Minato, Tokyo, Japan.; 3 Advanced Medical Research Center, Graduate School of Medicine, Yokohama City University, Yokohama, Kanagawa, Japan.; 4 Division of Immunology, Jiangsu Key Laboratory of Molecular Medicine, Medical School, Nanjing University, Nanjing, PR China.; 5 Department of Pathology, Immunology and Microbiology, Graduate School of Medicine, The University of Tokyo, Minato, Tokyo, Japan.; 6 Department of Clinical Pharmaceutics, Nihon Pharmaceutical University, Saitama, Japan.; 7 Division of Clinical Genome Research, Advanced Clinical Research Center, The Institute of Medical Science, The University of Tokyo, Tokyo, Japan.

**Keywords:** human induced pluripotent stem cells, liver organoids, microwell bags, scalable manufacturing, acute liver failure

## Abstract

**Rationale:**

Acute liver failure (ALF) is a life-threatening disease associated with high mortality rates. Human induced pluripotent stem cell (hiPSC)-derived liver organoids (LOs) hold considerable promise as cell-based bridging therapies for ALF. However, the scalable production of highly consistent and mature LOs remains a major barrier to their clinical translation. In this study, we aimed to establish a scalable manufacturing platform for the reproducible generation of highly uniform and functionally mature liver organoids.

**Methods:**

We generated LOs from hiPSCs and established a differentiation platform designed for high-density LO production using low-attachment culture vessels combined with spin culture. The hepatic functions of conventionally cultured LOs (Con-LOs) and spin-cultured LOs (Spin-LOs) were evaluated using qPCR, albumin secretion assay, and detoxification assay. The molecular effects of spin culture on LO development were further analyzed using RNA sequencing. Furthermore, microwell bags were introduced to enhance manufacturing scalability. Finally, the LOs were transplanted into the intraperitoneal spaces of acetaminophen (APAP)-induced ALF mice to assess their therapeutic potential.

**Results:**

Compared to Con-LOs, Spin-LOs exhibited markedly elevated hepatic gene expression and significantly enhanced liver-specific functions. Mechanistically, spin culture enabled a metabolic transition from glycolysis to oxidative phosphorylation, thereby driving further functional maturation of liver organoids, resulting in improved synthetic, metabolic, and detoxification capacities. Moreover, integrating a microwell bag-based LO formation system with spin-culture-driven LO differentiation further promoted the efficiency and scalability of functional LO manufacturing. Importantly, these LOs provided short-term metabolic support following intraperitoneal transplantation into immunocompetent mice and significantly ameliorated APAP-induced liver failure.

**Conclusion:**

This study establishes a scalable manufacturing platform for the reproducible generation of highly uniform and functionally mature liver organoids. Our findings provide an evidence-supported foundation for their future clinical translation as cell-based bridging therapies for ALF.

## Introduction

Acute liver failure (ALF) is a rapidly progressive and life-threatening syndrome characterized by the abrupt loss of essential hepatic metabolic, synthetic, and detoxification functions in patients without pre-existing liver disease. Owing to its rapid onset and narrow therapeutic window, ALF frequently progresses to coagulopathy, hepatic encephalopathy, and multiorgan failure, resulting in high short-term mortality 1-2. Currently, emergent liver transplantation remains the gold standard treatment for ALF and can markedly improve survival 3. However, the severe shortage of donor organs prevents patients from receiving timely treatment. Importantly, ALF is predominantly driven by acute functional insufficiency rather than irreversible structural damage. This characteristic highlights the need for therapies capable of rapidly restoring hepatic function to stabilize patients and bridge them to liver regeneration or transplantation 4-7.

Effective bridging therapy for ALF requires immediate provision of sufficient hepatic function including metabolic homeostasis, detoxification, and plasma protein synthesis. This approach must deliver a large mass of functionally mature hepatic cells within a short time frame, without the need for long-term tissue engraftment 4-7. However, the existing cell-based strategies are inadequate for meeting these requirements. Primary human hepatocytes (PHHs) are limited by donor scarcity, reduced viability after cryopreservation, and low engraftment efficiency 4. Hepatocyte-like cells generated by reprogramming often exhibit immature phenotypes and insufficient functional output 8-9. Extracorporeal bioartificial liver support systems provide only transient and limited benefits owing to the inability to sustain robust hepatic function *ex vivo* 10.

Human pluripotent stem cell (hPSC)-derived liver organoids have emerged as a promising platform for bridging therapy. Compared to hepatic cells generated by conventional two-dimensional (2D) differentiation protocols, liver organoids generally exhibit superior hepatic function 11-12. Recent studies have established scalable strategies for generating liver organoids from hPSC-derived expandable hepatoblasts, endodermal stem cells, and hiPSC aggregates 13-15. However, the expandable hepatoblast- and endodermal stem cell-based protocols rely on Matrigel for organoid formation and expansion, which limits their clinical application 13-14. On the other hand, Matrigel-free approaches depend on spontaneous organoid aggregation during dynamic culture. Consequently, the generated organoids often exhibit substantial variability in size and functional properties, which compromises the product consistency, quantitative evaluation, and reproducibility. Therefore, there is an urgent need for a manufacturing strategy that enables the scalable production of uniform and functionally mature liver organoids.

We have previously developed a self-organized LO system using human iPSC-derived hepatic endoderm cells (HEs), mesenchymal cells (MCs), and endothelial cells (ECs) in a microwell array platform. This system produced uniform multicellular LOs without relying on Matrigel 16. These LOs recapitulate multiple liver-specific functions related to metabolism, protein synthesis, and detoxification, supporting their potential use as a bridging therapy for ALF. However, clinical translation of this platform is limited by its manufacturing scalability. The restoration of approximately 5% of the total liver mass—corresponding to over 10^10 functional hepatocytes—is required to provide meaningful hepatic support in ALF 4. Meeting this requirement with our microwell-based method would require more than 2,000 culture plates for a single patient. This task would result in substantial labor requirements, manufacturing complexity, and increased batch-to-batch variability. Thus, increasing production scale without compromising organoid uniformity represents a key requirement for translating this platform toward clinical application.

In this study, we established a scalable manufacturing platform for producing highly uniform and functional human iPSC-derived LOs. By integrating controlled organoid assembly with prolonged spin-culture maturation, this platform simultaneously enabled scalable production, improved organoid uniformity, and enhanced hepatic maturation, thereby addressing key translation barriers in organoid-based therapy. Our platform provides a foundation for the efficient generation of functionally mature liver organoids and represents an important step toward their future clinical translation as a cell-based bridging therapy for ALF.

## Materials and Methods

### iPS cell culture

Human iPSC line M48 (CiRA Foundation) was used to generate hepatic endoderm, endothelial cells, and mesenchymal cells. hiPSCs were cultured on dishes coated with iMatrix-511 (Nippi; diluted 1:150 in phosphate-buffered saline [PBS]) in StemFit AK02N medium (Ajinomoto, AK02N) at 37 °C under humidified conditions with 5% CO₂. The medium was replaced every 2 days, and the cells were passaged at 7-day intervals using Accutase (Innovative Cell Technologies, AT104-500). The Rho kinase inhibitor Y-27632 (10 μM; Wako, 030-24026) was added to the culture medium for the first 24 h after cell seeding during both routine passage and differentiation.

### Liver organoid generation and culture

Human liver organoids were generated from hiPSCs according to a published protocol. The generation of hiPSC-derived hepatic endoderm cells (hiPSC-HEs), endothelial cells (ECs), and mesenchymal cells (MCs) was conducted according to a previously reported method. To generate LOs, hiPSC-HEs, hiPSC-ECs, and hiPSC-MCs in the ratio of 10:2:2 are co-cultured in microwell plates (Corning® Elplasia). For each well of a 24-well Elplasia plate, a total of 3.5 × 10^5^ cells (2.5 × 10^5^ hiPSC-HEs, 5 × 10^4^ hiPSC-ECs, and 5 × 10^4^ hiPSC-MCs) were seeded in 1 mL of culture medium, yielding approximately 500 LOs per well. Each LO initially contained approximately 700 cells, consisting of ~500 hiPSC-HEs, ~100 hiPSC-ECs, and ~100 hiPSC-MCs. For spin culture, organoids collected from two wells (approximately 1,000 LOs) were transferred into one well of a 6-well plate containing 2 mL of culture medium, thereby maintaining the same organoid density (approximately 500 LOs/mL). LOs are cultured in DMEM (Wako)/KBM-VEC1 basal medium (Kohjin Bio, Saitama, Japan) (DMEM: KBM = 1:1) supplemented with 2.5% fetal bovine serum (FBS), oncostatin M (10 ng/mL; R&D systems), dexamethasone (50 nM; Merck, Darmstadt, Germany). In addition, rotation speed for spin culture is 120 revolutions per minute (rpm). The selection of rpm was to maintain homogeneous suspension of liver organoids while minimizing excessive mechanical stress. The medium was changed daily during culture. The liver organoids were differentiated and maintained at 37 °C in a humidified incubator with 5% CO_2_.

### RNA isolation and quantitative polymerase chain reaction (qPCR)

Total RNA was isolated using a PureLinkTM RNA Mini Kit (Thermo Fisher Scientific, 12183025). Collected RNA (< 2 μg) was used as a template for single-strand cDNA synthesis with a high-capacity cDNA reverse transcription kit (Thermo Fisher Scientific, 4368814) according to the manufacturer's instructions. qPCR was performed using TB Green Premix Ex Taq II (Takara, RR820D) with a CFX96 Real-Time System machine (BioRad). The qPCR was conducted according to the manufacturer’s instructions. All data were calculated using the ΔΔCT method, with *ACTB* defined as the housekeeping gene. The primer sequence used in this study are listed in Table S1.

### Measurement of human albumin in supernatant

Supernatant was collected at certain time point after medium change for enzyme-linked immunosorbent assay (ELISA) using a human albumin ELISA quantification kit according to manufacturer’s instructions. Samples were diluted in a range from 20 to 500-fold to fit the linear range of standard curve.

### CYP3A4 enzyme activity

CYP3A4 activity was assessed using the CYP450-Glo Assay Kit (Promega, Madison). Briefly, organoids were incubated in fresh culture medium containing the luminogenic CYP substrate for 1 h at 37 °C. Following incubation, the culture supernatant was harvested, and the luciferin-based reaction and luminescence measurement were performed according to the manufacturer’s protocol.

### Ammonia elimination assay

Organoids were incubated for 24 h in RPMI 1640 medium (Wako, 030-24026) containing B-27™ (50×) (Gibco, 17504-001) and 2 mM NH4^+^. Ammonia concentrations in each well were measured at the beginning (C_0_) and after 24 h of incubation (C_24_) using a PocketChem BA Blood Ammonia Analyzer (Arkray) following the manufacturer’s protocol. Ammonia metabolism was determined using the following equation: ammonia metabolism = (C_0_ - C_24_)/C_0_ × V_Medium_.

### Urea production assay

The urea in supernatant was detected using Urea Assay Kit (Funakoshi, DIUR-100) according to the manufacturer’s instructions. The samples were diluted 50-fold to fit the standard curve.

### Cell number estimation and normalization

To estimate the total cell number of liver organoids, genomic DNA was extracted using the DNeasy Blood & Tissue Kit (Qiagen) according to the manufacturer's instructions. Organoids with known cell numbers on day 0 and organoids cultured on days 10 and 20 were collected, and genomic DNA was eluted in 50 μL of elution buffer. DNA concentration was measured using a NanoDrop spectrophotometer (Thermo Fisher Scientific). A standard relationship between DNA content and cell number was established using the day 0 samples with known cell numbers, and this calibration was subsequently used to estimate the total cell number of organoids on day 10 and 20. The estim ated cell numbers were used to normalize functional assays.

### Indocyanine green (ICG) uptake and release assay

For the ICG uptake assay, organoids were incubated with medium containing ICG (DAIICHI SANKYO, 2 mg/mL) for 4 h at 37 °C. After incubation, the organoids were collected, washed three times with PBS, and immediately examined under a microscope. For the ICG release assay, the organoids were subsequently incubated in fresh medium overnight at 37 °C to allow ICG release. Images of ICG uptake and release were acquired using a Nikon Eclipse TS100 microscope.

### Low-Density Lipoprotein (LDL) uptake assay

Organoid LDL uptake analysis was performed with LDL uptake Kit (Invitrogen, I34360) according to the manufacturer’s instruction. Then, the cells were washed with PBS, and the photographs were captured using a fluorescence microscopy.

### Hematoxylin & Eosin staining

Organoids were fixed in 4% paraformaldehyde for 15 min at room temperature and washed with PBS for three times. Before embedding in paraffin, fixed organoids were embedded with 2% low-melting point agarose gel solution (w/v in PBS). Liver tissues were fixed in 10% formalin solution (Wako, 066-03847) for at least 24 h. Paraffin blocks were cut in 5-μm section, and the sections were deparaffinized and dehydrated in xylene and serial concentration of ethanol (100%-50%) before staining in hematoxylin for 10 min. Samples were then washed with tap water for 30 min and stained with eosin for 10 min, following by dehydration with xylene and ethanol.

### Immunofluorescence staining

Tissue sections were deparaffinized & dehydrated with xylene and ethanol, and subjected to autoclave antigen retrieval in a 10 mmol/L citric acid buffer (pH = 6.0). The sections were then washed with PBS and blocked with a blocking buffer that consists of 10% ECL Prime Blocking Agent (Roche, RPN418) in PBS containing 0.3% Triton X-100 (Sigma, X100). Respective primary antibodies were diluted accordingly in the blocking buffer and incubated with samples at 4 °C overnight. The sections or cells were washed three times with PBS and incubated with corresponding secondary antibodies at a 1:500 dilution in the blocking buffer for another 60 min at room temperature. Finally, the cells were washed three times with PBS and covered with a mounting solution containing DAPI (1:1000). The antibodies used in this study are listed in Table S2. Fluorescence imaging was performed using a Leica Thunder or TCS SP8 confocal microscope.

### Transmission electron microscopy (TEM) sample preparation and imaging

Samples were fixed overnight at 4 °C in 0.1 M phosphate buffer (pH 7.4) containing 2% paraformaldehyde and 4% glutaraldehyde. After fixation, samples were postfixed in 2% osmium tetroxide, dehydrated with a graded ethanol series, and embedded in 100% resin. Ultrathin sections (70 nm) were prepared and stained with 2% uranyl acetate, followed by washing with distilled water and staining with a lead stain solution. The sections were examined using a JEM-1400Plus microscope, and images were acquired with an Olympus Veleta camera.

### Image presentation

All histological, functional staining, and ultrastructural analyses (including H&E staining, LDL uptake, ICG uptake/release assays, immunofluorescence staining, and transmission electron microscopy) were performed on multiple organoids from independent culture batches under identical experimental conditions. Representative images from randomly selected organoids are shown. Comparable morphological and functional patterns were consistently observed across examined samples.

### Bulk RNA sequencing analysis

Total RNA was prepared from indicated samples using a PureLinkTM RNA Mini Kit. RNA integrity was evaluated using Agilent 2100 Bioanalyzer (Agilent Technologies, Santa Clara, California), and RNA samples with RNA integrity number > 9.0 were subjected to RNA sequencing (RNA-Seq) analysis. RNA-Seq libraries were prepared using 100 ng of total RNA with an Ion AmpliSeq Transcriptome Human Gene Expression kit (Thermo Fisher Scientific, A26326) according to the manufacturer's instructions. The libraries were sequenced on an Ion Proton system using an Ion PI Hi-Q Sequencing 200 kit and Ion PI Chip v3 (Thermo Fisher Scientific, A26772), and the sequencing reads were aligned to hg19_AmpliSeq_Transcriptome_ERCC_v1 using Torrent Mapping Alignment Program. Subsequently, QC metrics and normalized read counts per gene were obtained using AmpliSeqRNA plug-in v5.2.0.3, a Torrent Suite Software v5.2.2 (Thermo Fisher Scientific). Data were analyzed using GeneSpring (Agilent Technologies). Gene ontology enrichment and pathway analysis were performed on DAVID Bioinformatics Resources 6.8 (https://david.ncifcrf.gov/home.jsp). Gene Set Enrichment Analysis was performed and plotted with a tool from the Broad Institute (GSEA v4.3.2). Heatmaps were created with GeneSpring and Graphpad Prism 9.

### Glucose uptake assay

The concentration of glucose in supernatant (C_1_) and fresh medium (C_2_) were measured by Glucose Metabolism Assay (Dojindo, G264) according to manufacturer’s instructions. The glucose uptake was calculated as follow: (C_2_-C_1_)/C_2_×100%.

### Adenosine triphosphate (ATP) production assay

The cellular ATP was measured by ATP Assay Kit-Luminescence (Dojindo, A550) according to manufacturer’s instructions.

### Lactate production assay

The concentration of lactate in supernatant was measured by Lactate Assay Kit-WST (Dojindo, L256) according to manufacturer’s instructions.

### Analysis of the activities of Phase I and II metabolic enzymes

Organoids were cultured in RPMI1640 supplemented with a combination of metabolic substrates (1/100 dilution) at 37 °C for 1 hr. Then, the culture supernatants were harvested, and metabolites were quantified by LC-MS/MS as described previously with minor modifications. The substrates were a gift from Professor Yasuhiro Yamada from Nihon Pharmaceutical University. The substrates included specific compounds that are metabolized by different Phase I and II metabolic enzymes. The list of substrates and their respective enzymatic pathways are as follows: 40 μM phenacetin (a CYP1A2 substrate), 50 μM bupropion (a CYP2B6 substrate), 0.1 μM amodiaquin (a CYP2C8 substrate), 5 μM diclofenac (a CYP2C9 substrate), 100 μM S-mephenytoin (a CYP2C19 substrate), 5 μM bufuralol (a CYP2D6 substrate), 5 μM midazolam (a CYP3A substrate), and 100 μM 7-hydroxycoumarin (a UGT and SULT substrate).

### Liver organoids generated in large-sized microwell bag

One day before cell seeding, the bag was half-filled with medium (200 mL), attached to the jig, and incubated at 37 °C, 95% humidity, and 5% CO_2_ atmosphere for overnight to remove the microbubbles. Large bubbles were removed by syringes through the port. On seeding day, another 200 mL medium containing a mixture of 2×10^8^ HEs, 4×10^7^ ECs, and 4×10^7^ MCs was injected into the bag. After removing air in the bag, cells were mixed well by gently tapping the bag. Finally, the bag was reattached to the jig and incubated at 37 °C for 24 h for organoid formation.

### Liver organoids collection from microwell bag

24 h after seeding day, liver organoids were formed in microwell bags. By gentle inversion, organoids were suspended in the medium. The bag was hung up, and organoids were harvested. The harvested organoids were then equally allocated into 4 lipidure-coated 250 mL flasks on orbital shaker for culture.

### Blood biochemical analysis

Retro-orbital blood samples were collected from mice before and after transplantation for biochemical analysis. The level of Aspartate Transaminase (AST), Alanine Aminotransferase (ALT), Total bilirubin (T-Bil), Direct bilirubin (D-Bil), blood ammonia, and blood sugar in serum were monitored using FUJI DRI-CHEM SLIDE by FUJI DRI-CHEM analyzer FDC7000VZ.

### Induction of ALF and transplantation of LOs in mice

Male C57BL/6 mice (6–8 weeks old) were purchased from Japan SLC Inc. (Tokyo, Japan). Mice were housed under standard conditions at the University of Tokyo Animal Center with a 12 h light/dark cycle, an ambient temperature of 22 ± 2 °C, and a relative humidity of 50 ± 10%. To establish the ALF model, mice were first fasted for 24 h to deplete hepatic glutathione stores and enhance susceptibility to APAP-induced liver injury. APAP (Sigma, A5000-100G) was dissolved in saline and administered intraperitoneally at a dose of 350 mg/kg body weight. 16 h after APAP administration (overnight), LOs generated using the microwell bag platform were harvested on culture day 20, washed twice with PBS, and transplanted into the peritoneal cavity of recipient mice. LOs were suspended in 200 μL PBS and transplanted intraperitoneally using a 1 mL syringe fitted with a 27G needle. In the experimental timeline, the time of LO transplantation was defined as 0 h; therefore, APAP administration and fasting initiation corresponded to -16 h and -40 h, respectively. All animal experiments were performed according to the ethical rules established by The Institute of Medical Science, The University of Tokyo animal experiment committee (PA22-05).

### Temporal dynamic of metabolic pathways

Pathway analysis of the bulk RNA seq data (Figure 5F) was performed as follows: the expression of each gene comprising selected Gene Oncology Biological Process (GOBP) pathways was normalized by division by the maximal expression level across the 12 groups (each timepoint has two duplicates). Then, the expression score of one group for certain pathway was the mean of all the gene expression. Those means were further normalized to the maximal mean-expression across 12 groups for each pathway.

### Quantification analysis

The percentage of necrotic area, number of positive cells, or percentage of positive area, of H&E staining or immunostaining images was quantified with ImageJ. Three fields of view per mouse, from different lobes, were analyzed and averaged.

### Use of AI-assisted tools

OpenAI was used solely for grammar checking and language refinement during manuscript preparation. It was not used for data generation, image generation or modification, data analysis, or scientific interpretation.

### Figure preparation

Schematic illustrations included in the figures and supplementary materials were created using BioRender.

### Statistics

GraphPad Prism 9.0 (GraphPad Software) was used for statistical analysis. No data was excluded from the data analysis. The Mann-Whitney test was used, and *p* < 0.05 was considered statistically significant (*P < 0.05; **P < 0.01; ***P < 0.001, ****P < 0.0001, ns: not significant). The *in vitro* studies were conducted in at least three biological replicates. For *in vivo* studies, mice were randomly divided into experimental groups, with at least three mice per group. The number of mice used was determined using the minimum necessary animals to ensure no waste of animal life. Study-size determination was influenced by the robustness of the biological endpoint. All data analyses were performed unblind without any exclusions of animals.

## Results

### Establishment of an efficient and scalable pattern for LO differentiation

To overcome the limitations of conventional culture systems for the large-scale production of functional LOs, we investigated whether spin culture, a scalable platform widely used in cell manufacturing 13-15, could support LO maturation while preserving organoid uniformity. Building on our previously established protocol 16, LOs were first assembled in microwell array plates by co-culturing hiPSC-HEs, hiPSC-ECs, and hiPSC-MCs. After 24 h, 1,000 LOs were transferred to a 6-well plate containing 2 mL of culture medium and cultured on an orbital shaker at 120 rpm (Figure 1A and Figure S1A). However, under these conditions, spin culture proved to be ineffective. Within the first 3 h, the number of free-floating organoids decreased sharply, with only 23.5 ± 7.2% remaining in suspension (Figure 1B), and by 24 h, organoids were nearly undetectable in the culture vessel. Notably, most of the missing organoids adhered tightly to the walls of the culture vessel (Figure S1B), indicating that organoid–surface adhesion was the major cause of spin culture failure. To overcome this issue, the entire inner surface of the culture vessel was coated with 0.5% Lipidure to generate an ultra-low-attachment surface. Following this modification, over 90% of the organoids remained freely suspended after 24 h (Figure 1B-C).

We characterized the morphological and structural features of LOs after 10 days of *in vitro* differentiation. Spin-cultured LOs (Spin-LOs) maintained a well-defined morphology and exhibited a comparable size to that of conventionally cultured LOs (Con-LOs) during the culture (Figure 1D-E). Histological analysis by hematoxylin and eosin (H&E) staining revealed that cells within Spin-LOs were densely packed with a more compact tissue organization. In contrast, Con-LOs displayed a looser cellular arrangement (Figure 1F). Transmission electron microscopy (TEM) demonstrated that Spin-LOs possessed well-organized hepatocyte ultrastructure, including clearly defined tight junctions and bile canaliculi, indicating advanced epithelial polarity and structural maturation (Figure 1G).

At the molecular level, quantitative polymerase chain reaction (qPCR) analysis showed that Spin-LOs exhibited significant upregulation of hepatic function–related genes, including albumin (*ALB*), cytochrome P450 enzymes (*CYP3A5*, *CYP2C9*, *CYP7A1*, and *CYP3A4*), and key urea-cycle genes (*OTC* and *CPS1*), compared to Con-LOs (Figure 1H). Consistently, immunofluorescence staining further confirmed the extensive expression of corresponding hepatic markers throughout Spin-LOs, supporting enhanced hepatocytic differentiation (Figure 1I). Functionally, Spin-LOs exhibited a marked enhancement of core hepatic activities consistent with the upregulation of these genes. Compared to Con-LOs, Spin-LOs demonstrated an approximately 6-fold increase in albumin secretion, a 15-fold increase in CYP3A4 enzymatic activity, and a 60% improvement in ammonia clearance capacity (Figure 1J). Spin-LOs displayed a broader spectrum of mature hepatocyte functions, including efficient indocyanine green (ICG) uptake and release as well as low-density lipoprotein (LDL) uptake, indicating the global functional maturation of hepatocyte-like cells (Figure S1C-D). These findings showed that spin culture improves hepatic differentiation and function of LOs, with changes observed at the molecular, structural, and functional levels.

Next, we investigated the effects of organoid density and rotational speed on the spin culture of the LOs. Organoids were cultured at densities of 250, 500, or 1,000 LOs/mL. By day 10, no clear differences were observed among the three groups in morphology, size, or albumin secretion (Figure S1E–G). However, the expression of hepatic maturation markers, including *CYP3A4* and *OTC*, was significantly reduced in the 1,000 LOs/mL group compared to the 250 and 500 LOs/mL groups, suggesting that excessively high organoid density may impair hepatic maturation (Figure S1H). To optimize the rotational speed, we compared rotational speeds of 60 and 120 rpm. Notably, organoid aggregation was observed at 60 rpm after 9 days, whereas organoids cultured at 120 rpm remained uniformly suspended and maintained their morphology throughout the culture period (Figure S1I). Based on these optimization experiments, an organoid density of 500 LOs/mL and a rotational speed of 120 rpm were selected as the standard conditions for all subsequent spin-culture experiments.

### Spin culture promoted hepatic maturation through a metabolic switch

Next, we investigated the molecular basis of the effects of spin culture on LO development. Transcriptomic analysis revealed that both Con-LOs and Spin-LOs progressively upregulated the expression of hepatic function–related genes during differentiation (Figure S2A). Despite this common change, principal component analysis (PCA) demonstrated two separate differentiation trajectories, indicating a progressive divergence in developmental programs (Figure 2A). Gene Ontology (GO) analysis of the differentially expressed genes (DEGs) also showed differences between the two culture conditions (Figure S2B). On day 7, Spin-LOs showed significant enrichment of gene sets associated with cell cycle progression and DNA replication, whereas Con-LOs were predominantly enriched in HIF-1 signaling, an indicator of hypoxia. By day 10, pathways related to the tricarboxylic acid (TCA) cycle and pyruvate metabolism were enriched in Spin-LOs, whereas HIF-1 signaling remained prominent in Con-LOs (Figure 2B). These transcriptomic patterns suggested that spin culture drives fundamental metabolic reprogramming during LO differentiation. Under static conditions, activation of hypoxia-associated signaling pathways such as HIF-1 promoted glycolytic metabolism and suppresses mitochondrial oxidative metabolism 17-18. In comparison, the improved oxygen availability under spin culture may support mitochondrial metabolism and promote a metabolic transition from glycolysis toward oxidative phosphorylation.

Analysis of enzyme-coding genes involved in glycolysis and the TCA cycle showed clear differences between the two culture conditions. Con-LOs exhibited significantly higher expression of glycolysis-related genes, whereas Spin-LOs showed enhanced expression of TCA cycle–associated genes (Figure 2C). Metabolic switching plays an important role in cell fate decisions and development 19. Cells modulate their metabolism in response to variations in environmental and physiological conditions. We therefore performed functional assays to examine the metabolic state of the LOs. Although glucose consumption was comparable between the two groups, Spin-LOs produced significantly lower levels of lactate and exhibited significantly higher intracellular ATP levels. These results suggested enhanced mitochondrial oxidative metabolism in Spin-LOs (Figure 2D-F). Consistent with these observations, immunofluorescence staining and TEM revealed increased mitochondrial density in Spin-LOs compared to Con-LOs, accompanied by significant upregulation of mitochondrial-encoded genes (*ND1–ND6, CO1–CO3, CYB*) (Figure 2G-I).

To assess whether mitochondrial metabolism contributes to Spin-LO maturation, we pharmacologically modulated the TCA cycle. Spin-LOs were treated with Devimistat 20, an inhibitor of pyruvate dehydrogenase (PDH) and α-ketoglutarate dehydrogenase (KGDH). This treatment reduced the expression of mitochondrial and CYP genes and decreased CYP3A4 activity (Figure 2J-K). Conversely, enhancement of mitochondrial metabolism in Con-LOs using GSK2837808A 21, an inhibitor of lactate dehydrogenase, induced significant upregulation of mitochondrial and CYP genes and increased CYP3A4 activity (Figure 2L-M). Collectively, these results demonstrated that spin culture induces a metabolic shift from glycolysis to mitochondrial oxidative metabolism. They also suggested that the metabolic changes induced by spin culture contribute to the enhanced hepatic function of Spin-LOs.

### Prolonged culture augmented organoid maturation under spin-culture conditions

Given that spin culture enhanced TCA cycle activity and promoted hepatic maturation, we next investigated whether prolonged culture under spin-culture conditions could further enhance organoid maturation (Figure 3A). Spin-LOs maintained a stable size and well-defined morphology after an additional 10 days of culture. Histological analysis by H&E staining revealed a densely organized hepatic tissue architecture, whereas TEM demonstrated ultrastructural features characteristic of mature hepatocytes, including a reduced nuclear-to-cytoplasmic ratio and polygonal cell shapes with prominent apical microvilli (Figure 3B).

To investigate temporal changes in molecular identity, we compared the transcriptomic profiles of Spin-LOs on day 1, day 10, and day 20, using primary human hepatocytes (PHHs) as positive controls. Although Spin-LOs did not fully reach the transcriptional levels observed in PHHs, they exhibited a clear shift toward an adult-like hepatocyte program (Figure 3C). Adult hepatocyte markers, including *HP* and *ADH1B* 22, were highly expressed in day-20 Spin-LOs. Clustering analysis and Gene Set Enrichment Analysis (GSEA) revealed that genes upregulated on day 20 were significantly enriched in liver-specific functions such as xenobiotic metabolism, lipid metabolism, bile acid synthesis, and complement activation. Concomitantly, cell cycle–related gene expression was markedly downregulated, indicating a transition from a proliferative immature state to a more differentiated phenotype (Figure S3A-B). Quantitative PCR analysis further confirmed a dramatic reduction in the fetal liver marker *AFP*, accompanied by a significant upregulation of key hepatic functional genes such as *ALB*, *OTC*, *CPS1*, and CYP family members on day 20. Compared to PHHs, day-20 Spin-LOs exhibited lower expression of *CPS1* and drug-metabolizing CYP genes (*CYP3A4*, *CYP2B6*, *CYP3A5*, and *CYP2C9*), despite their significant upregulation during prolonged spin culture (Figure 3D). Consistent with these transcriptional changes, prolonged spin culture markedly enhanced hepatic function. Although several hepatic functions remained below the levels observed in PHHs, albumin secretion, CYP3A4 activity, ammonia clearance, and urea production increased significantly between day 10 and 20 (Figure 3E). Liquid chromatography–mass spectrometry further demonstrated significantly elevated Phase I (CYP3A, CYP2B6, CYP2C9, and CYP2D6) and Phase II (UGT)-mediated drug metabolism in day-20 Spin-LOs (Figure 3F). Immunofluorescence staining also supported the enhanced hepatic maturation of day-20 Spin-LOs, with a notable increase in CPS1 expression (Figure 3G). Con-LOs maintained their morphology and histological architecture during prolonged culture. Their albumin secretion and urea production increased modestly, but the increases were not as great as those observed in Spin-LOs (Figure S4A-C). Ammonia clearance and drug-metabolizing activities showed no further improvement in Con-LOs during extended culture (Figure S4C-D). Collectively, these findings indicated that prolonged spin culture supported progressive hepatic maturation more effectively than conventional static culture, resulting in greater improvements in both hepatic synthetic and metabolic functions.

We further evaluated Spin-LOs during extended culture for up to 50 days to explore the functional ceiling of LOs. Spin-LOs maintained their structural integrity throughout the culture period, with no apparent apoptosis or dissociation (Figure S5A). Immunofluorescence analysis confirmed the maintenance of hepatic characteristics during this period (Figure S5B). However, hepatic function gradually declined after day 20. This was reflected by lower *ALB* expression, reduced albumin secretion, and decreased CYP3A4 activity from day 20 to day 50 (Figure S5C-E). Collectively, these findings demonstrated that spin culture substantially promoted LO maturation during prolonged culture, with day 20 representing the optimal time point for achieving peak metabolic and detoxification capacities. Therefore, day-20 Spin-LOs were selected for subsequent therapeutic evaluation.

### Scale-up manufacturing of functional LOs

Despite the substantial improvement in functional maturation by spin culture, the clinical translation of LOs for ALF therapy requires high functional performance as well as the availability of a large number of organoids. Currently, LO generation relies on conventional microwell plates, which are inherently limited in scalability. A single microwell plate produces only approximately 17,000 organoids per batch, which makes it impractical to generate the millions of organoids required for therapeutic use. Moreover, plate-based systems operate in open culture environments, which increases the risk of contamination during cell seeding and harvesting. Therefore, scalable and standardized organoid generation remains a critical bottleneck for translating functionally mature LOs into clinically applicable products. To overcome these limitations, we introduced a microwell bag system capable of simultaneously generating up to 650,000 organoids (Figure S6A) 23. With this optimization, only approximately 30 microwell bags were required to achieve the same production scale as 2,000 microwell plates. Furthermore, the estimated collection time would be reduced from approximately 200 h to 5 h, accompanied by substantial reductions in culture space and labor requirements. Collectively, these improvements markedly enhanced the overall manufacturing efficiency and scalability of LO production (Figure S6B).

Next, we integrated the microwell bag system with spin culture to evaluate its feasibility for large-scale LO manufacturing (Figure 4A). Similar to organoids derived from microwell plates (Plate-LOs), the hiPSC-HEs, hiPSC-ECs, and hiPSC-MCs seeded in microwell bags could also self-organize into organoids (Bag-LOs). Bag-LOs maintained a well-defined morphology and structural stability throughout 20 days of spin culture (Figure 4B-C). qPCR analysis demonstrated that day-20 Bag-LOs expressed hepatic function–related genes, including *ALB*, *G6PC*, *CPS1*, *OTC*, and CYP450 family members, which were comparable to the levels observed in Plate-LOs (Figure 4D). Day-20 Bag-LOs exhibited synthesis and detoxification functions, such as albumin secretion, CYP3A4 activity, ammonia clearance, and urea production (Figure 4E-H), with strong expression of the corresponding markers (Figure 4I). Taken together, these findings demonstrated that the combination of microwell bags and spin culture enabled efficient and scalable manufacturing of functional LOs while reducing the labor and operational costs associated with organoid production, thereby providing a practical manufacturing platform for future clinical translation.

### Intraperitoneal transplantation of functional LOs rescues immunocompetent mice from APAP-induced ALF

Given the rapid progression of ALF in clinical settings, an easy-to-use, allogeneic organoid transplantation strategy is crucial for effective intervention. First, we evaluated the therapeutic potential of LOs. Bag-LOs harvested on day 20 were transplanted into the peritoneal cavity of immunocompetent mice (Figure S7A), a minimally invasive approach that provides temporary support of liver functions 24. Serial monitoring of human albumin levels in mouse serum revealed that the transplanted LOs remained functionally active within 3 days after transplantation (Figure S7B). We also evaluated the safety of LO transplantation. Bioluminescence imaging showed that the signal of luciferase-labeled LOs gradually declined within 7 days, and no abnormal proliferative foci were observed during the 6-month follow-up period (Figure S7C). Furthermore, histological examination using H&E and Sirius Red staining revealed no evidence of tumor formation, abnormal tissue growth, or fibrosis, supporting the preliminary long-term safety of intraperitoneal LO transplantation (Figure S7D). To assess the local immune response, the transplanted LOs were collected from the peritoneal cavity 24 h after transplantation. Immunofluorescence staining revealed infiltration of macrophages (F4/80^+^ and CD68^+^) and neutrophils (Ly6G^+^) within the transplanted LOs (Figure S8A-B). However, the serum levels of IL-1β, IL-6, and TNF-α did not significantly increase in transplanted mice (Figure S8C). The recollected LOs maintained their hepatic characteristics, with Albumin, CYP3A4, and CPS1 clearly detected by immunofluorescence staining (Figure S8D).

Next, we evaluated the therapeutic efficacy of the Bag-LOs in an APAP-induced ALF model (Figure 5A) 25. Administration of APAP (350 mg/kg) induced fulminant liver injury and 71% mortality (29 of 38 mice), establishing a stringent experimental model of ALF. Intraperitoneal transplantation of day-20 Bag-LOs containing 3 × 10⁶ hepatic cells, corresponding to approximately 3% of total liver mass, significantly improved survival, rescuing 66.7% (16 of 24 mice) from ALF compared with untreated controls (Figure 5B). Consistently, LO-treated mice exhibited an accelerated recovery of body weight (Figure 5C). Human albumin (Figure 5D) and human coagulation factors V, XII, and XIII were also detected in the LO-transplanted mice (Figure S9A), indicating that the transplanted LOs retained liver-specific synthetic function *in vivo*. Meanwhile, the levels of liver injury indicators, aspartate transaminase (AST) and alanine aminotransferase (ALT), were significantly reduced in the transplantation group (Figure 5E). In addition to transaminase levels, both LO-treated and control mice exhibited a progressive decline in serum bilirubin and ammonia levels, accompanied by a partial recovery of blood glucose after APAP injury (Figure S9B). To determine whether the therapeutic efficacy depends on functional maturation status, we additionally transplanted day-10 Bag-LOs generated under identical spin culture conditions at the same cell dose. In contrast to day-20 Bag-LOs, day-10 Bag-LOs failed to significantly improve survival, and serum AST and ALT levels remained comparable to those in the sham group, indicating limited hepatoprotective capacity at this early maturation stage (Figure S10A-B). These findings suggested that the therapeutic benefits in the ALF model required greater functional maturity.

To further characterize the regenerative process following LO transplantation, transcriptomic profiling was performed at early time points after APAP injury. LO transplantation was associated with earlier activation of regeneration-related pathways 26, including focal adhesion, WNT signaling, Hippo signaling, and angiogenesis, within 24 h, whereas similar pathway activation was observed only at 48 h in the control livers (Figure 5F). Consistent with these transcriptomic findings, histological analyses revealed accelerated hepatic regeneration in the LO-treated mice. At 24 h after APAP administration, extensive hepatocellular damage affected approximately half of the liver parenchyma in both groups. Only a limited number of Ki67⁺ hepatocytes were detected in the residual regions. From 24 to 48 h, liver tissue recovered more rapidly in LO-transplanted mice. H&E staining showed a greater restoration of the liver parenchyma, and Ki67 staining revealed an increased hepatocyte proliferation (Figure 5G-J).

APAP-induced hepatotoxicity is typically accompanied by acute sterile inflammation and immune cell recruitment. We therefore analyzed the immune cell populations in injured livers following LO transplantation. Consistent with previous reports 27, we found that Kupffer cells (F4/80^high^ CD68^low^) were markedly reduced in injured regions after APAP administration. In contrast, infiltrating monocytes and monocyte-derived macrophages (F4/80^low^ CD68^high^) accumulated in damaged areas and represented the dominant responding population for debris clearance and tissue remodeling (Figure 5K-N and Figure S11A). Previous studies have suggested that monocyte-derived macrophages can negatively regulate neutrophils during tissue injury and repair 27. At 48 h after APAP administration, the transplantation group showed more monocyte-derived macrophages than the sham group. This increase was accompanied by a reduction in Ly6G⁺ neutrophils (Figure S11B-C). Collectively, these findings demonstrated that intraperitoneally transplanted functional LOs rescued immunocompetent mice from APAP-induced ALF while accelerating liver regeneration and remodeling the liver immune microenvironment.

## Discussion

The persistent shortage of donor livers for transplantation highlights the urgent need for alternative therapeutic strategies that provide rapid and effective hepatic functional support. Our previous studies have demonstrated that hiPSC-derived LOs possess robust hepatic functions *in vitro* and have therapeutic potential *in vivo* 16, 28-29. Building on these findings, we addressed a critical bottleneck in the translation of liver organoid-based therapies by establishing a scalable manufacturing platform for the reproducible generation of highly uniform and functionally mature liver organoids. Compared to conventional approaches, this system supports large-scale production of LOs and promotes hepatic maturation, as indicated by enhanced protein synthesis, ammonia clearance, urea production, and drug-metabolizing capacities. More importantly, the integration of microwell bags and spin culture substantially reduced the labor requirements, culture space, and equipment demands for organoid generation, collection, and differentiation, thereby improving the manufacturing efficiency and standardization. LOs produced using this platform provided hepatic functional support after intraperitoneal transplantation and improved survival in immunocompetent mice with APAP-induced ALF. Overall, this study provides a scalable approach for producing functional LOs and supports their further development as a potential therapy for ALF.

Maintaining uniformity, reproducibility, and quality control during scale-up is a major challenge in translating organoid technologies into clinical applications. Previously reported spin-cultured organoids were highly heterogeneous with substantial variability in size, cellular composition, and functional properties 13-15. Such heterogeneity compromises product consistency, quantitative evaluation, and reproducibility of manufacturing processes. In this study, we addressed this challenge by using a two-layer quality control strategy. First, LOs were pre-assembled in microwell platforms to control their initial size and cellular composition. Second, culture vessels were modified with a low-attachment surface to reduce organoid adhesion and aggregation during spin culture. This helped maintain a uniform morphology and distribution throughout the culture period. Together, these complementary strategies enabled scalable production while preserving organoid uniformity, reproducibility, and functional maturation. The resulting system provides a more standardized approach for large-scale LO production and may facilitate future clinical translation.

Notably, our findings indicate that spin culture exerts a profound influence on the maturation trajectory of LOs. Compared to static culture, spin culture may improve the availability of oxygen and nutrients to meet the increasing metabolic demands of differentiating hepatic lineages. Consistent with this notion, Spin-LOs exhibited downregulation of HIF-1 hypoxic signaling and a metabolic shift from glycolysis toward the TCA cycle, accompanied by enhanced mitochondrial biogenesis, suggesting progression toward a more mature hepatic state 30. Similar functional declines have been reported in cultured PHHs and liver organoids 31-32. One possible explanation is the lack of a native liver microenvironment, including appropriate cell–cell interactions, tissue organization, and biochemical signals. Among the pathways implicated in this process, TGF-β signaling has also been associated with hepatocyte dysfunction and senescence and can suppress several metabolic functions 31, 33-34. Consistent with these reports, our RNA-seq analysis detected sustained expression of TGF-β in liver organoids during prolonged culture, suggesting that persistent TGF-β signaling may contribute to the gradual decline in hepatic function observed after day 20. However, the precise mechanisms of long-term functional deterioration remain to be elucidated.

Hepatocyte transplantation is typically performed via the portal vein, spleen, or the peritoneal cavity 35. Compared to portal or splenic delivery, intraperitoneal transplantation offers unique advantages for organoid-based therapy. The large peritoneal cavity provides sufficient space for delivering a large number of organoids and does not require vascular access. This is particularly important for our LOs. The relatively large size of LOs (about 200 μm) may increase the risk of portal hypertension and severe bleeding, and the peritoneal route offers a safer alternative. Notably, a recent clinical study reported that intraperitoneal transplantation of encapsulated hepatocytes in eight children with ALF led to four natural recoveries and three successful bridges to liver transplantation 36, supporting the safety and efficacy of intraperitoneal transplantation of hepatic lineages. Intraperitoneal transplantation often relies on encapsulation strategies to protect transplanted cells from immune rejection and enhance their survival 36-37. In contrast, here, we directly transplanted LOs into the peritoneal cavity of immunocompetent mice without encapsulation.

Based on the detection of human-specific proteins together with *in vivo* imaging, we found that the transplanted LOs exhibited essential hepatic functions in the first three days, which resulted in a significant amelioration of liver injury and regeneration, indicating that transient hepatic functional support is critical for liver recovery from ALF. During this period of transient hepatic functional support, the transplanted LOs retained liver-specific synthetic activity *in vivo*, as evidenced by the detection of human albumin and coagulation factors (V, XII, and XIII) in mouse serum. Albumin is a multifunctional plasma protein that maintains plasma oncotic pressure and plays essential roles in molecular transport, antioxidant defense, detoxification, and immune regulation 38. During ALF, loss of albumin synthesis contributes to metabolic dysfunction and impaired systemic homeostasis. Therefore, albumin secreted by the transplanted LOs may provide temporary metabolic and synthetic support during the critical phase of liver injury. Furthermore, previous studies have suggested that certain coagulation factors, including factor XIII, may participate in liver regeneration 39. Taken together, these findings support the hypothesis that transplanted LOs provide transient hepatic functional support *in vivo*. However, the relative contributions of individual secreted factors, and the potential involvement of paracrine signaling, remain to be elucidated.

The present platform effectively addresses the scalable manufacturing of functional liver organoids, whereas the current large-scale generation of hiPSC-derived HEs, ECs, and MCs relies on direct differentiation in conventional 2D culture systems, which require substantial labor, culture media, growth factors, and manufacturing resources. Therefore, establishing robust and scalable expansion systems for these cell populations, together with further optimization of culture media and manufacturing processes, will be an important step toward reducing production costs and enabling the clinical-scale manufacturing of liver organoids. Additionally, translating the current research-grade protocol into a xeno-free, GMP-compatible manufacturing process, including the replacement of FBS-containing culture conditions, is essential for the clinical translation of this technology.

## Conclusions

In this study, we established an efficient and scalable platform for large-scale production of functional liver organoids. This system holds significant promise for therapeutic applications and may contribute to *in vitro* research such as high-throughput drug screening, toxicity testing, and disease modeling. Moreover, the platform can be readily extended to generate other types of organoids to meet the growing demands. Future applications require further development of scalable upstream cell production, xeno-free and GMP-compatible culture systems, and standardized manufacturing processes to ensure consistent quality, safety, and efficacy.

## Supplementary Material

Supplementary figures and tables.

## Figures and Tables

**Figure 1:**
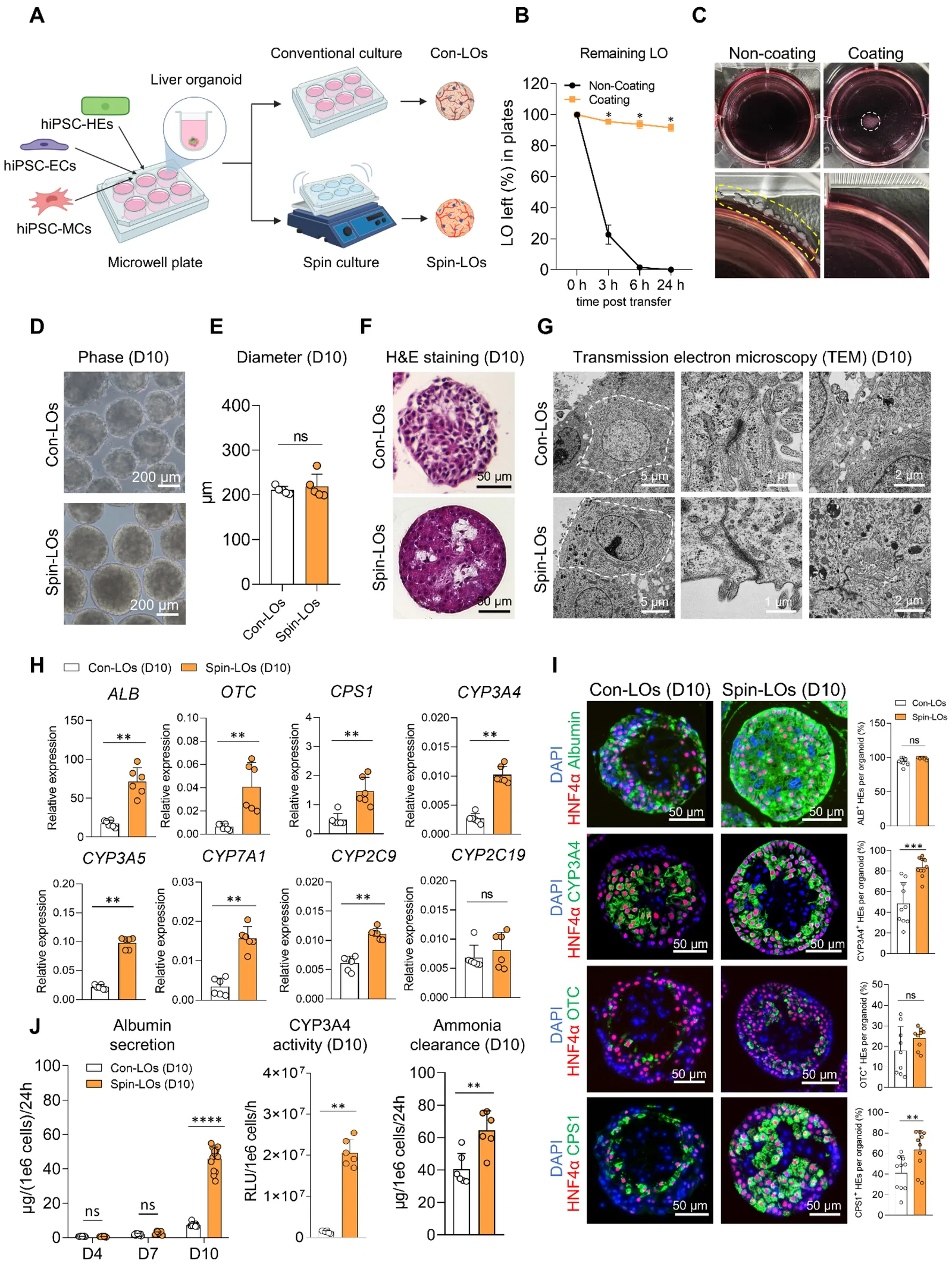
** Establishment of an efficient and scalable platform for LO differentiation.** A Schematic representation illustrating the orbital shaker-based spin culture system for LOs in 6-well plates and LOs in conventional microwell plates. B Change of number of remaining LOs in one well over time after transfer into 6-well plates (n = 4). C Image of wells with and without 0.5% Lipidure coating. White dotted line indicates LOs in suspension. Yellow dotted line indicates adhering LOs to the surface. D Phase images depicting the Con-LOs and Spin-LOs on day 10. E Long diameter of LOs on day 10 (n = 5). Each dot represents an average diameter of one batch (organoid number > 200). F H&E staining of LOs on day 10. G TEM image showing the ultrastructure of LOs. White dotted line indicates hepatic cells. H Expression of hepatic functional genes (*ALB*, *OTC*, *CPS1*), and cytochrome P450 superfamily (*CYP3A5*, *CYP2C9*, *CYP2C19*, *CYP7A1*, *CYP3A4*) of LOs on day 10 (n = 6). I Left: Immunofluorescence staining of Albumin, CYP3A4, OTC and CPS1 with hepatic marker HNF4α of LOs on day 10. Right: statistics of hepatic cells positive for specific marker (n = 9). J Function analysis of albumin secretion (n = 14), ammonia elimination (n = 6), and CYP3A4 activity (n = 6) of LOs on day 10. All data are shown as mean ± S.D. Statistical analyses were performed using Mann-Whitney test. *P < 0.05, **P < 0.01, ***P < 0.001, ****P < 0.0001, ns: not significant.

**Figure 2:**
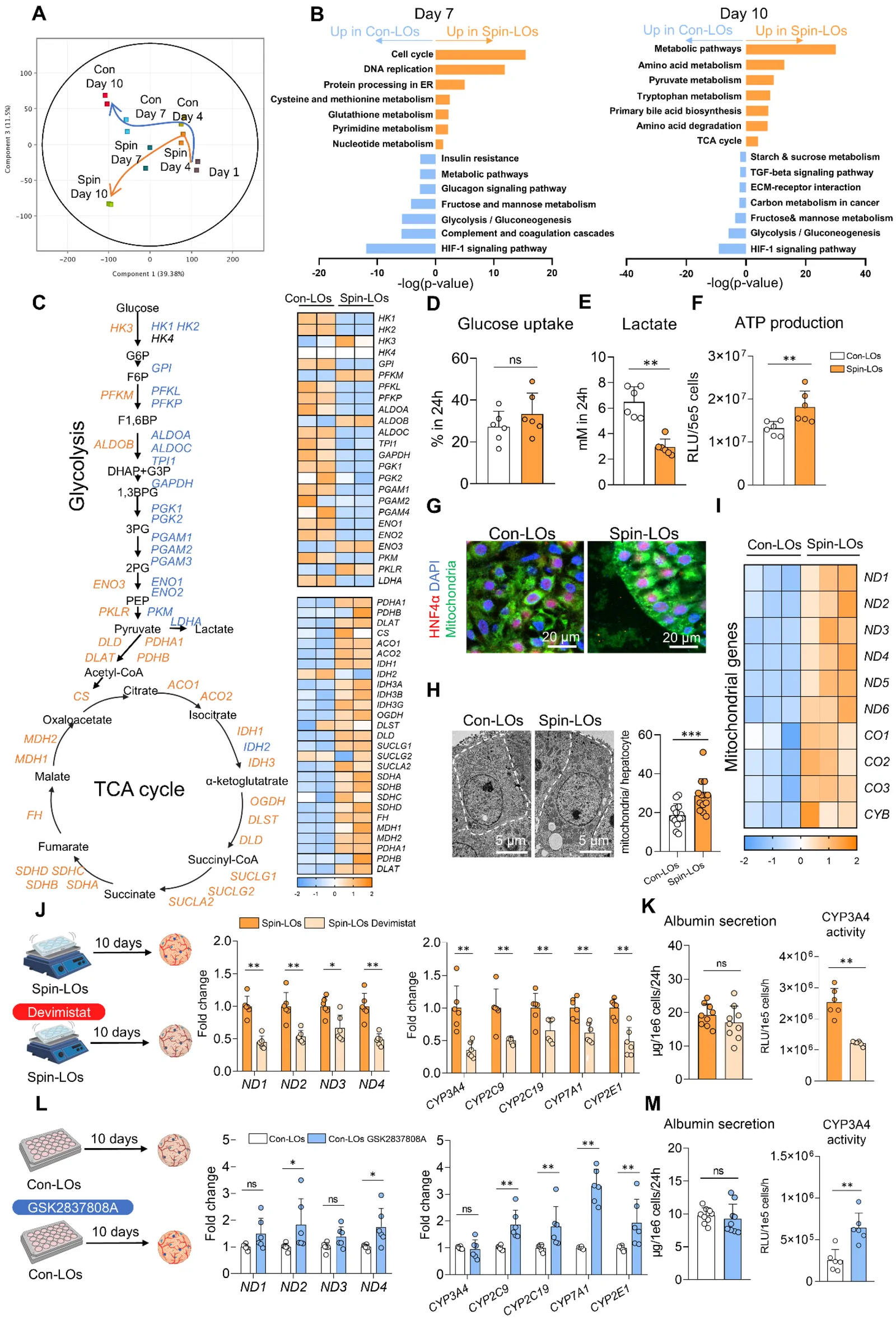
** Spin culture promoted hepatic maturation through a metabolic switch.** A Principal-co mponent analysis of bulk RNA sequencing of LOs on day 1, 4, 7 and 10 (n = 2). B GO analysis of DEGs of LOs on day 7 and day 10. C Left panel represents the pathway of glycolysis and TCA cycle. Genes in blue indicate higher expression in Con-LOs. Genes in orange indicate higher expression in Spin-LOs. Right panel represents the heatmap comparing the expression of genes involved in glycolysis and TCA cycle of Con-LOs and Spin-LOs on day 10 (n = 2). (G6P: Glucose-6-phosphate; F6P: Fructose-6-phosphate; F1, 6BP: Fructose-1,6-bisphosphate; DHAP: Dihydroxyacetone phosphate; G3P: Glyceraldehyde-3-phosphate; 1,3-BPG: 1,3-Bisphosphoglycerate; 3PG: 3-Phosphoglycerate; 2PG: 2-Phosphoglycerate; PEP: Phosphoenolpyruvate). D Glucose uptake by LOs in 24 h on day 10 (n = 6). E Lactate secretion into the culture medium of LOs incubated for 24 h (n = 6). F Intracellular ATP quantification in LOs (n = 6). G The co-immunofluorescent staining of mitochondria and HNF4α in LOs. H Left panel represents the ultrastructure of hepatic cells in LOs. Right panel represents the quantification analysis of mitochondria per hepatic cell (n = 13). I Heatmap comparing the expression of mitochondrial genes of LOs on day 10 (n = 3). J Schematic diagram showing Devimistat inhibition. Gene expression of mitochondria and cytochrome P450 superfamily of Spin-LOs with Devimistat after culture for 10 days (n = 6). K Albumin secretion (n = 9) and CYP3A4 activity (n = 6) upon Devimistat inhibition. L Schematic diagram showing GSK2837808A stimulation. Gene expression of mitochondria and cytochrome P450 superfamily of Con-LOs with GSK2837808A after culture for 10 days (n = 6). M Albumin secretion (n = 9) and CYP3A4 activity (n = 6) upon GSK2837808A stimulation. All data are shown as mean ± S.D. Statistical analyses were performed using Mann-Whitney test. *P < 0.05, **P < 0.01, ***P < 0.001, ****P < 0.0001, ns: not significant.

**Figure 3:**
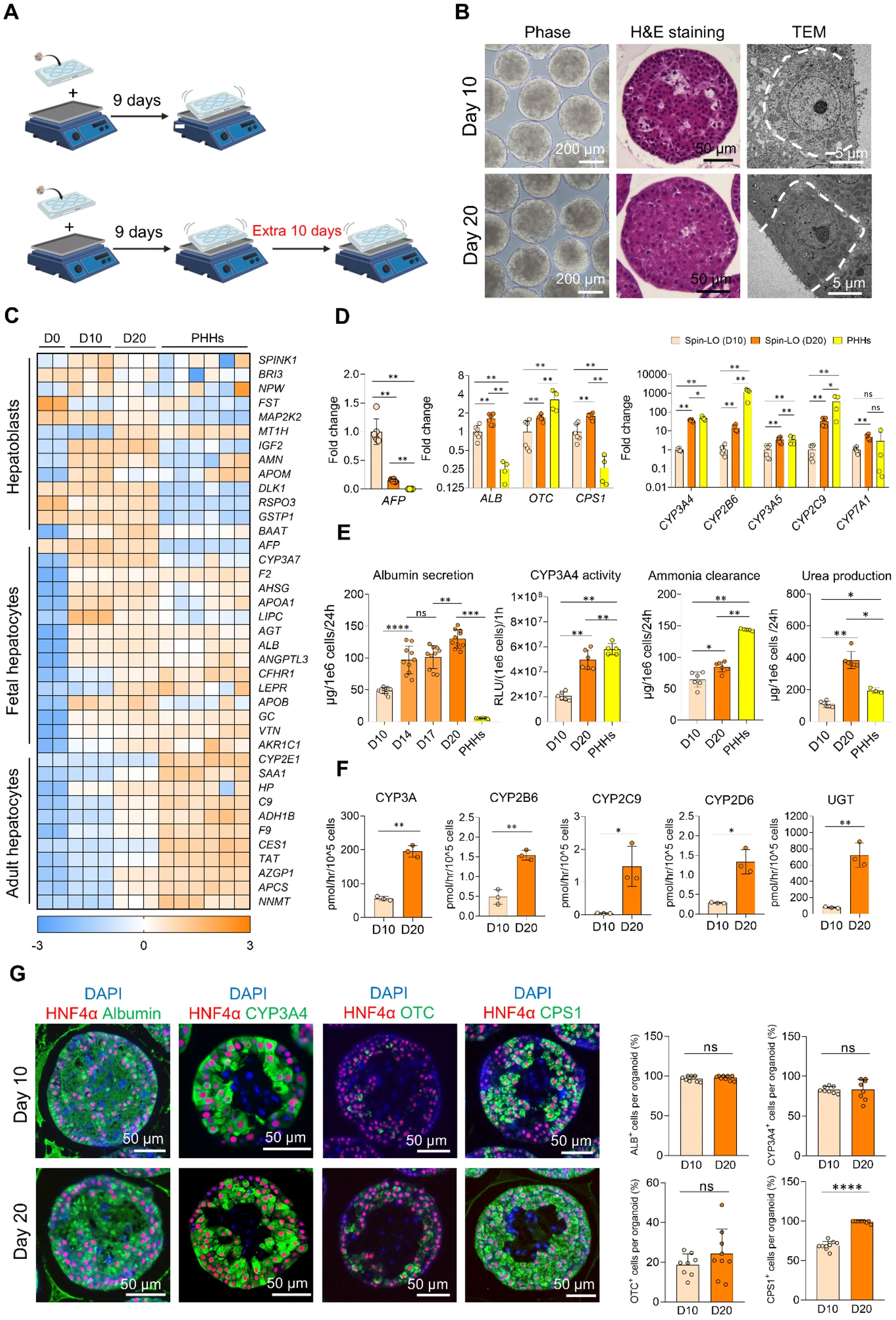
** Liver organoids further maturated under spin condition after prolonged culture.** A Schematic diagram showing the experimental design of extending culture period for LOs under spin condition. B Phase images, H&E staining, and TEM images of Spin-LOs on day 10 and day 20. C Heatmap of expression of signature genes during hepatocyte maturation of LOs from day 1 to day 20 and PHHs (day 1: n = 2; day 10: n = 3; day 20: n = 3; PHHs: n = 6). D Gene expression of fetal liver marker (AFP), functional markers (*ALB*, *G6PC*, *CPS1*), and cytochrome P450 superfamily (*CYP3A4*, *CYP2B6*, *CYP3A5*, *CYP2C9*, *CYP7A1*) of PHHs (n = 4) and Spin-LOs on day 10 and day 20 (n = 6). E Functional analysis of albumin secretion (Spin-LOs, n = 10; PHHs, n = 5), CYP3A4 activity (Spin-LOs, n = 6; PHHs, n = 5), ammonia clearance (Spin-LOs, n = 6; PHHs, n = 5), and urea production (Spin-LOs, n = 6; PHHs, n = 3) in day-10 and day-20 Spin-LOs and PHHs. F Drug metabolism assay of phase I and phase II enzymes of Spin-LOs on day 10 and day 20 (n = 3). G Left: Immunofluorescence co-staining of hepatic marker HNF4α and function markers (Albumin, CYP3A4, OTC, CPS1). Right: statistics of hepatic cells positive for specific marker (n = 9). All data are shown as mean ± S.D. Statistical analyses were performed using Mann-Whitney test. *P < 0.05, **P < 0.01, ***P < 0.001, ****P < 0.0001, ns: not significant.

**Figure 4:**
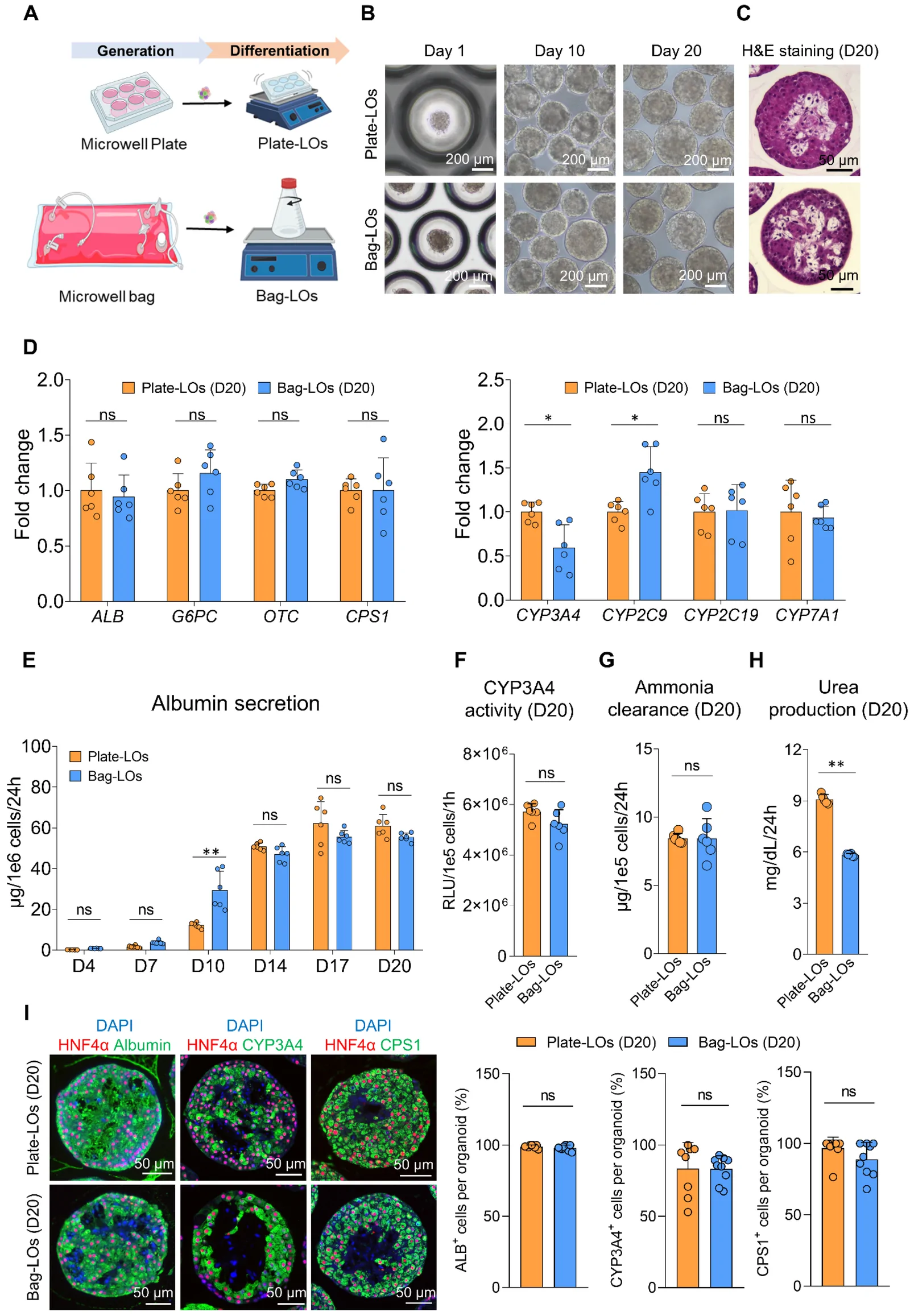
** Scale-up manufacturing of functional LOs.** A Schematic diagram representing the experimental design. LOs generated from microwell plates were cultured in 6-well plates. LOs generated from microwell bags were cultured in 250 mL flasks. B Phase images of Plate-LOs and Bag-LOs on day 1, day 10, and day 20. C H&E staining images of Plate-LOs and Bag-LOs on day 20. D Expression of hepatic functional genes (*ALB*, *G6PC*, *OTC*, *CPS1*) and Cytochrome P450 superfamily (*CYP3A4*, *CYP2B6*, *CYP3A5*, *CYP2C9*, *CYP7A1*) of Plate-LOs and Bag-LOs on day 20 (n = 6). Fold change was normalized to Plate-LOs. E Albumin secretion in supernatant of Plate-LOs and Bag-LOs on day 20 (n = 6). F Function assay of CYP3A4 activity of Plate-LOs and Bag-LOs on day 20 (n = 6). Fold change was normalized to Plate-LOs. G Function assay of ammonia clearance of Plate-LOs and Bag-LOs on day 20 (n = 6). Fold change was normalized to Plate-LOs. H Function assay of urea production of Plate-LOs and Bag-LOs on day 20 (n = 6). Fold change was normalized to Plate-LOs. I Left: Immunofluorescence co-staining of hepatic marker HNF4α with functional markers on day 20 (Albumin, CYP3A4, CPS1). Right: statistics of hepatic cells positive for specific marker (n = 9). All data are shown as mean ± S.D. Statistical analyses were performed using Mann-Whitney test. *P < 0.05, **P < 0.01, ***P < 0.001, ****P < 0.0001, ns: not significant.

**Figure 5:**
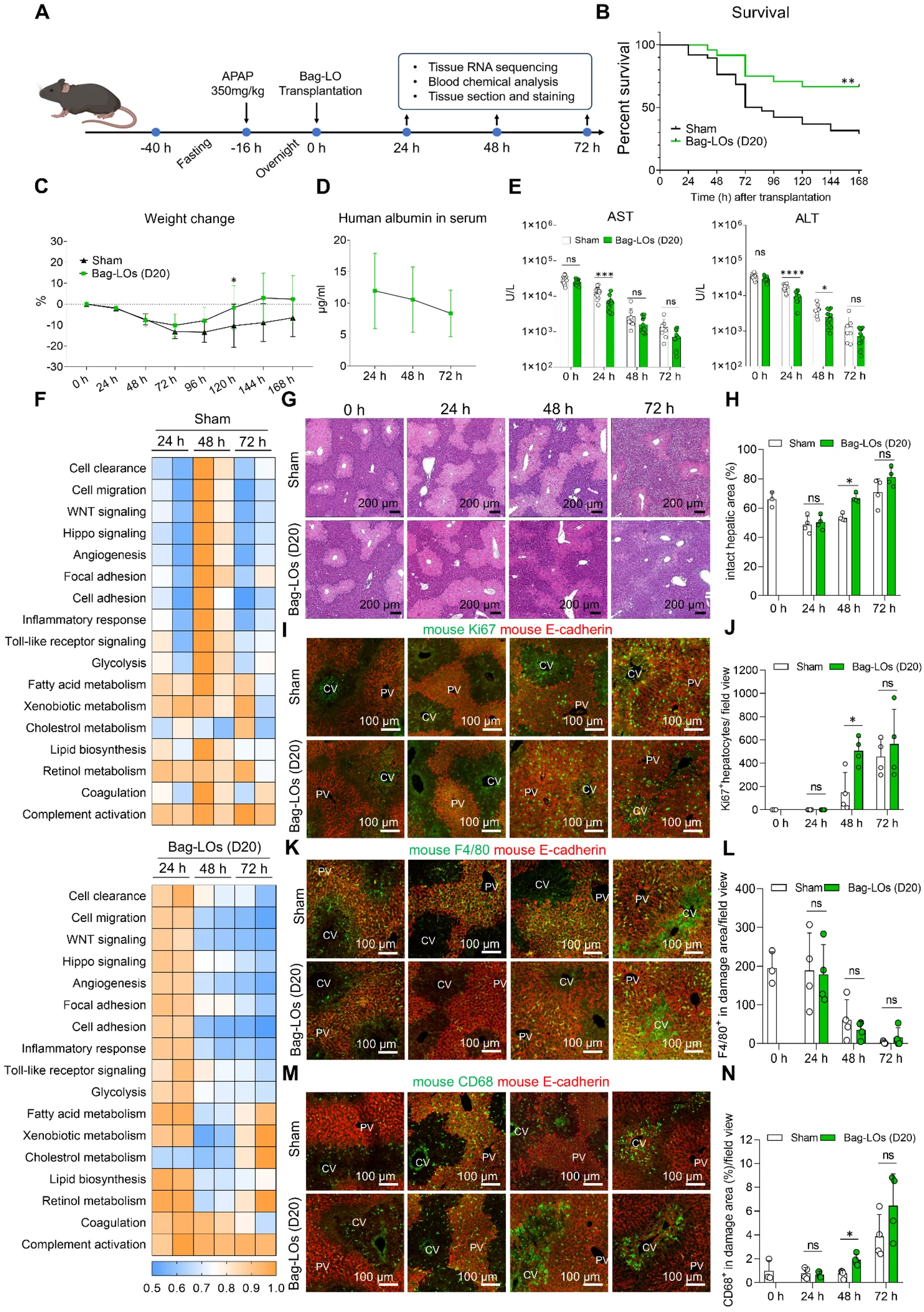
** Intraperitoneal transplantation of functional LOs rescued immunocompetent mice from APAP-induced ALF.** A Schematic diagram illustrating the induction of ALF model and intraperitoneal transplantation of day-20 Bag-LOs. B Kaplan-Meier survival curves of ALF mice transplanted with saline (Sham, n = 38) and Bag-LOs containing 3×10^6^ HEs (Bag-LOs, n = 24). C Weight change of mice during the first 7 days after transplantation (n = 17). D The amount of human albumin detected in the sera of mice after transplantation (n = 17). E Serum biochemical parameters measurements (AST, ALT) of mice after transplantation (Sham: n =15, Bag-LOs: n = 14). F Selected GOBP pathways which show temporal changes after transplantation, ranked by their time point of peak expression. G H&E staining of liver of the mice (n = 2). H Quantification analysis of necrotic area of liver based on H&E staining (0h: n = 3, 24-72h: n = 4). I Immunofluorescence co-staining of mouse Ki67 and E-cadherin of liver of the mice. J Quantification analysis of cells double positive for Ki67 and E-cadherin in defined areas (0h: n = 3, 24-72h: n = 4). K Immunofluorescence co-staining of mouse F4/80 and E-cadherin of mice liver after transplantation. L Quantification analysis of F4/80^+^ratio in necrotic area (0h: n = 3, 24-72h: n = 4). M Immunofluorescence co-staining of mouse CD68 and E-cadherin of mice liver after transplantation. N Quantification analysis of CD68^+^ ratio in necrotic area (0h: n = 3, 24-72h: n = 4). All data are shown as mean ± S.D. Statistical analyses were performed using Mann-Whitney test. Survival curve was analyzed using Log-rank test. *P < 0.05, **P < 0.01, ***P < 0.001, ****P < 0.0001, ns: not significant.

## Data Availability

All data supporting the findings of this study are available within the article and its additional information files. All materials are available on request.

## Abbreviations

ALF: Acute liver failure
LO: Liver organoid
hiPSC: Human induced pluripotent stem cell
APAP: Acetaminophen
Con-LO: conventionally cultured liver organoid
Spin-LO: spin cultured liver organoid
HEs: Hepatic endoderm cells
ECs: Endothelial cells
MCs: Mesenchymal cells
FBS: Fetal bovine serum
RPM: Revolutions per minute
ELISA: Enzyme-Linked Immunosorbent Assay
ICG: Indocyanine green
LDL: Low density lipoprotein
RNA-seq: RNA sequencing
ATP: Adenosine triphosphate
AST: Aspartate Aminotransferase
ALT: Alanine Transaminase
T-Bil: Total bilirubin
D-Bil: Direct bilirubin
CYP: Cytochrome P450
ALB: Albumin
PCA: Principal components analysis
GO: Gene ontology
DEG: Differentially expressed gene
TCA: Tricarboxylic acid
PDH: Pyruvate dehydrogenase
KGDH: Alpha-ketoglutarate dehydrogenase
PHH: Primary human hepatocyte
GSEA: Gene set enrichment analysis
Plate-LO: Microwell plate cultured liver organoid
Bag-LO: Microwell bag cultured liver organoid
