# Supplementary material for: Scalable manufacturing of human iPSC-derived functional liver organoids for acute liver failure treatment
Source: Theranostics. 2026 Sep 18;16(16):9277–94. doi: 10.7150/thno.137714 (PMC13684818; doi:10.7150/thno.137714)
Supplement: Supplementary file 1 [file thnov16p9277s1.pdf]

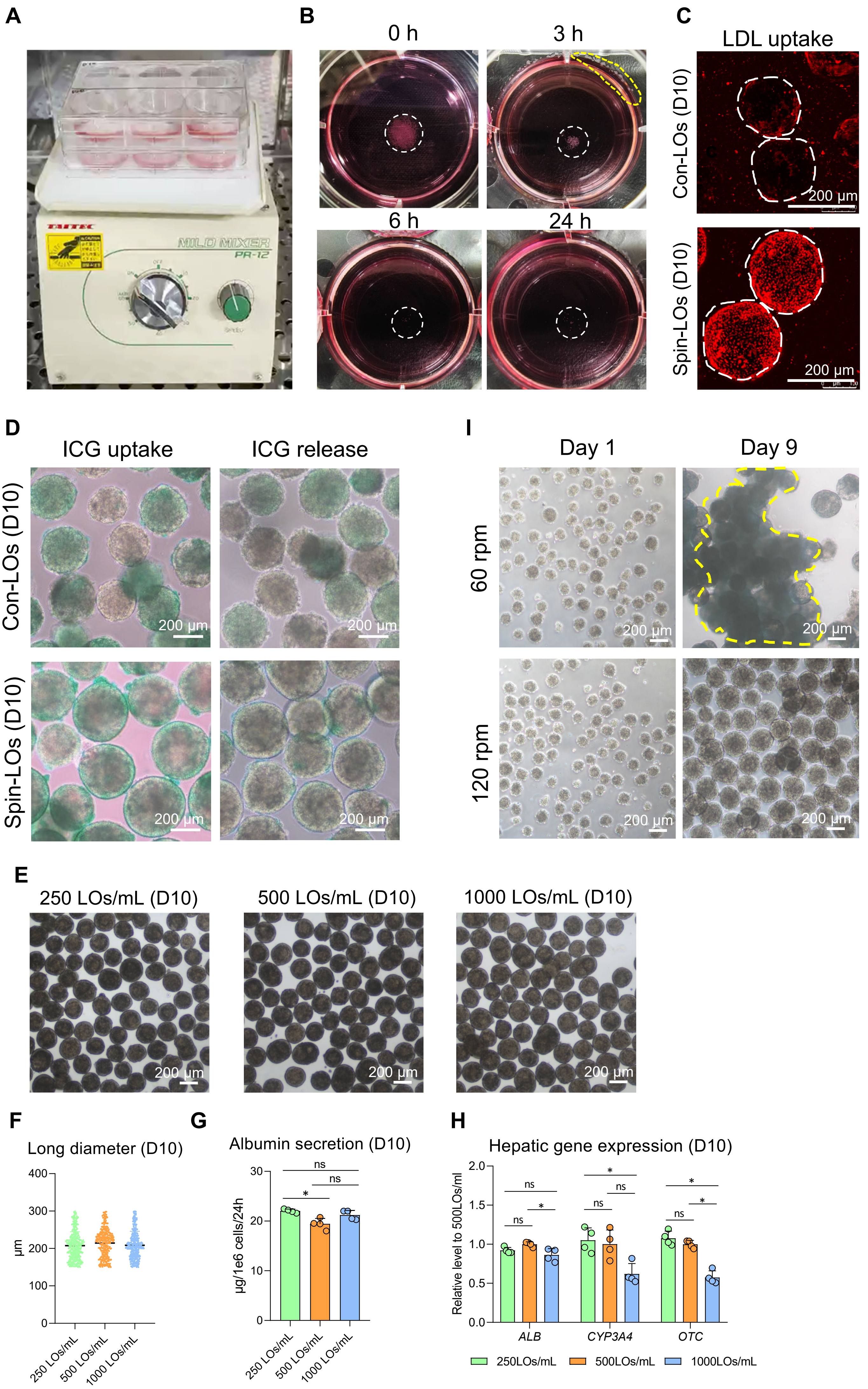

A
MILD MIXER
PR-12
B
0 h
3 h
6 h
24 h
C
LDL uptake
Con-LOs (D10)
Spin-LOs (D10)
200 μm
D
ICG uptake
ICG release
Con-LOs (D10)
Spin-LOs (D10)
200 μm
I
Day 1
Day 9
60 rpm
120 rpm
200 μm
E
250 LOs/mL (D10)
500 LOs/mL (D10)
1000 LOs/mL (D10)
200 μm
F
Long diameter (D10)
μm
400
300
200
100
0
250 LOs/mL
500 LOs/mL
1000 LOs/mL
G
Albumin secretion (D10)
μg/1e6 cells/24h
30
20
10
0
ns
ns
*
250 LOs/mL
500 LOs/mL
1000 LOs/mL
H
Hepatic gene expression (D10)
Relative level to 500LOs/ml
2.0
1.5
1.0
0.5
0.0
ns
*
*
ALB
CYP3A4
OTC
250LOs/mL
500LOs/mL
1000LOs/mL

Figure S1: Supplementary validation of spin culture system and LO functional assays.

A Image showing the orbital shaker-based spin culture system. **B** Images showing the gradual loss of suspension LOs in regulate culture wells. White dotted lines indicate the LOs in suspension. **C** LDL uptake of Con-LOs and Spin-LOs on day 10. **D** ICG uptake & release of Con-LOs and Spin-LOs on day 10. **E** Phase images of Spin-LOs at density of 250 LOs/mL, 500 LOs/mL, and 1,000 LOs/mL. **F** Long diameter of Spin-LOs at different density (n > 300). **G** Albumin secretion of Spin-LOs at different density (n = 4). **H** Hepatic gene expression of Spin-LOs at different density (n = 4). **I** Images showing the morphology of LOs at different rpm. Yellow dash line indicates the LO aggregates.

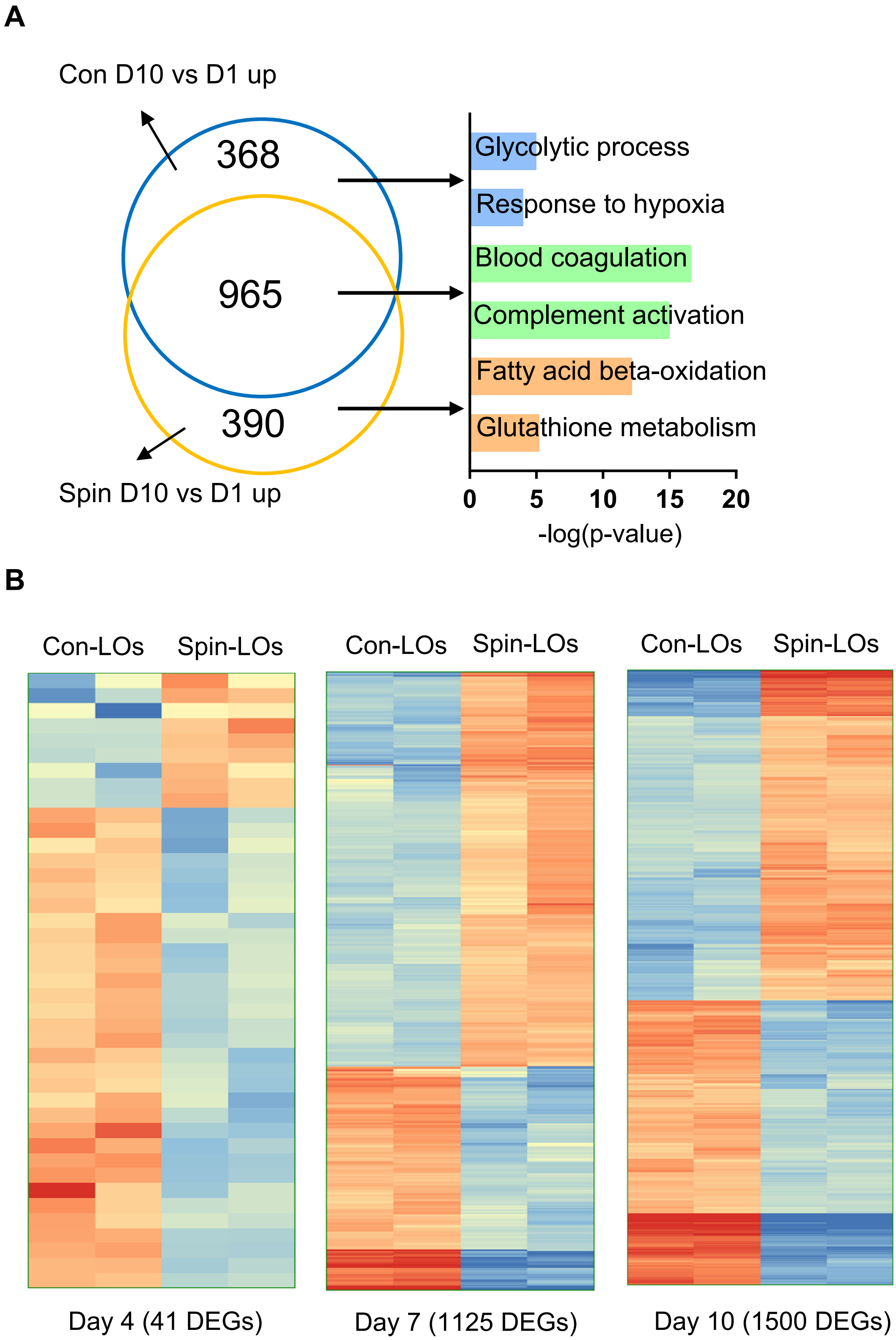
A
Con D10 vs D1 up
368
965
390
Spin D10 vs D1 up
Glycolytic process
Response to hypoxia
Blood coagulation
Complement activation
Fatty acid beta-oxidation
Glutathione metabolism
0
5
10
15
20
-log(p-value)
B
Con-LOs
Spin-LOs
Con-LOs
Spin-LOs
Con-LOs
Spin-LOs
Day 4 (41 DEGs)
Day 7 (1125 DEGs)
Day 10 (1500 DEGs)

Figure S2: Supplementary data of transcriptomic analysis of Con-LOs and Spin-LOs.

A Venn diagram of the upregulated genes in Spin-LOs and Con-LOs on day 10 compared to common day 1. Blue circle represents the upregulated genes in Con-LOs. Orange circle represents the upregulated genes in Spin-LOs. **B** Heatmap of DEGs of Con-LOs and Spin-LOs on day 4, 7, and 10 (n = 2).

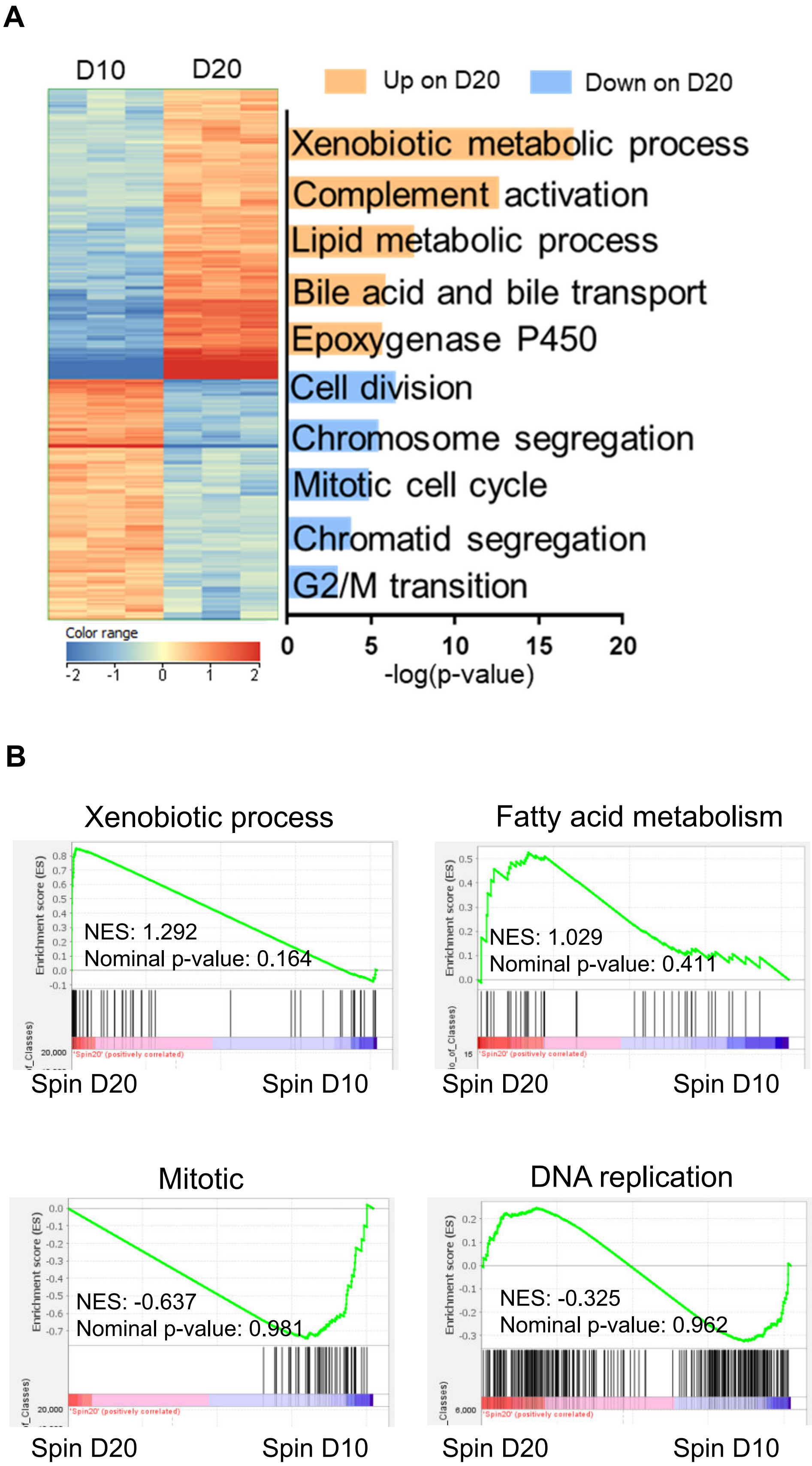
A
D10
D20
Up on D20
Down on D20
Xenobiotic metabolic process
Complement activation
Lipid metabolic process
Bile acid and bile transport
Epoxygenase P450
Cell division
Chromosome segregation
Mitotic cell cycle
Chromatid segregation
G2/M transition
Color range
-2 -1 0 1 2
0 5 10 15 20
-log(p-value)
B
Xenobiotic process
NES: 1.292
Nominal p-value: 0.164
Spin D20
Spin D10
Fatty acid metabolism
NES: 1.029
Nominal p-value: 0.411
Spin D20
Spin D10
Mitotic
NES: -0.637
Nominal p-value: 0.981
Spin D20
Spin D10
DNA replication
NES: -0.325
Nominal p-value: 0.962
Spin D20
Spin D10

Figure S3: Transcriptomic comparison between D10 and D20 Spin-LOs reveals enhanced metabolic programs and reduced cell cycle activity.

A Heatmap and pathway enrichment analysis of differentially expressed genes between day 10 and day 20 Spin-LOs (*n* = 2). **B** GSEA analysis between day 10 and day 20 Spin-LOs.

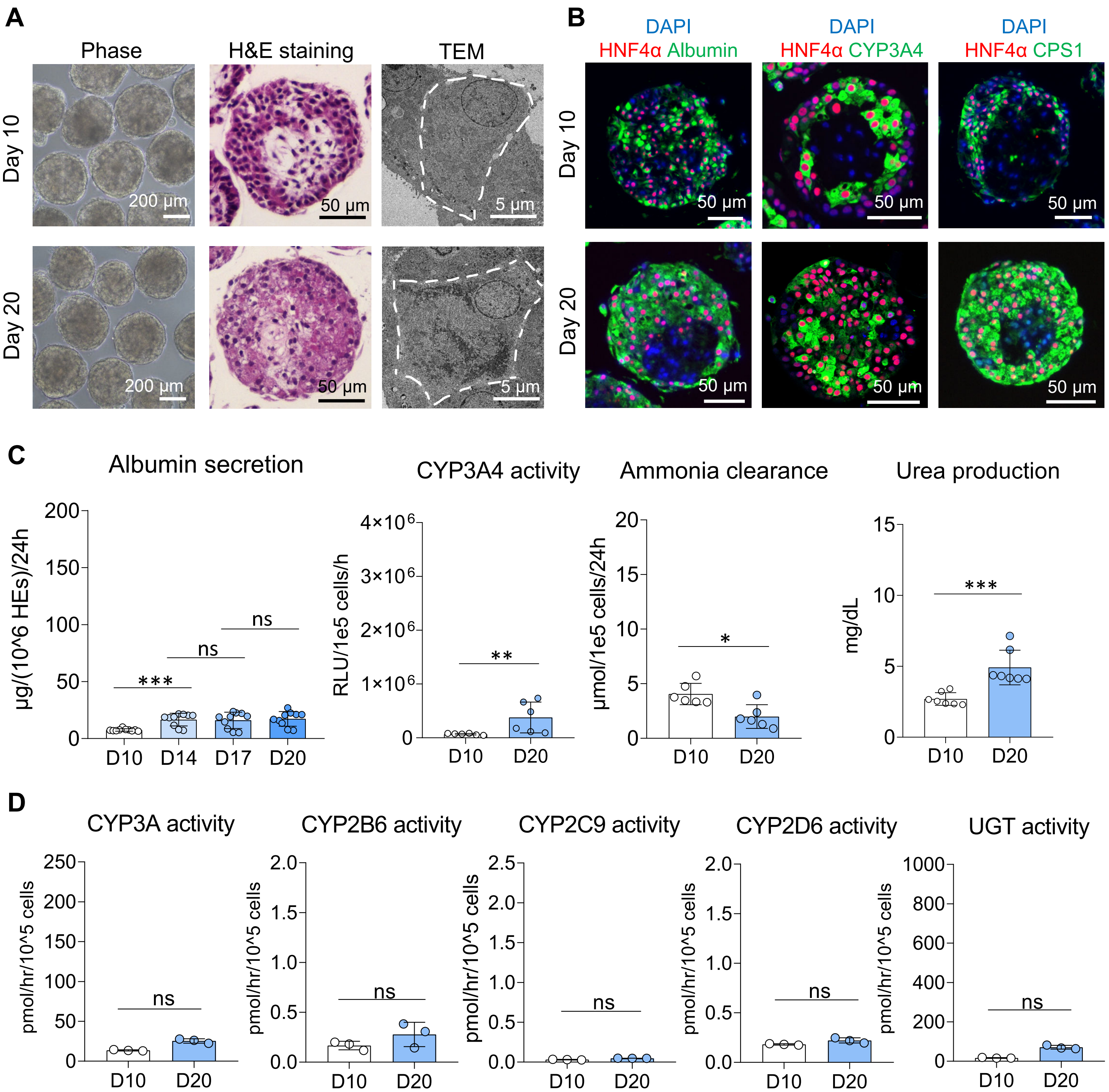

A
Phase
H&E staining
TEM
Day 10
Day 20
200 μm
50 μm
5 μm
B
DAPI
HNF4α Albumin
HNF4α CYP3A4
HNF4α CPS1
50 μm
C
Albumin secretion
μg/(10^6 HEs)/24h
D10
D14
D17
D20
***
ns
CYP3A4 activity
RLU/1e5 cells/h
**
Ammonia clearance
μmol/1e5 cells/24h
*
Urea production
mg/dL
***
D
CYP3A activity
CYP2B6 activity
CYP2C9 activity
CYP2D6 activity
UGT activity
pmol/hr/10^5 cells
ns

Figure S4: Prolonged culture organoid within conventional static condition.

A Phase images, H&E staining, ultrastructure of hepatic cells of Con-LOs on day 10 and day 20. **B** Immunofluorescence co-staining of hepatic marker HNF4α with functional markers and structural markers (Albumin, CYP3A4, CPS1) on day 20. **C** Function analysis of albumin secretion (n = 10), CYP3A4 activity (n = 6), ammonia clearance (n = 6), and urea production (n = 7) of Con-LOs on day 10 and day 20. **D** Drug metabolism assay of phase I and phase II enzymes of Con-LOs on day 10 and day 20 (n = 3). All data are shown as mean ± S.D. Statistical analyses were performed using Mann-Whitney test. *$P < 0.05$, **$P < 0.01$, ***$P < 0.001$, ns: not significant.

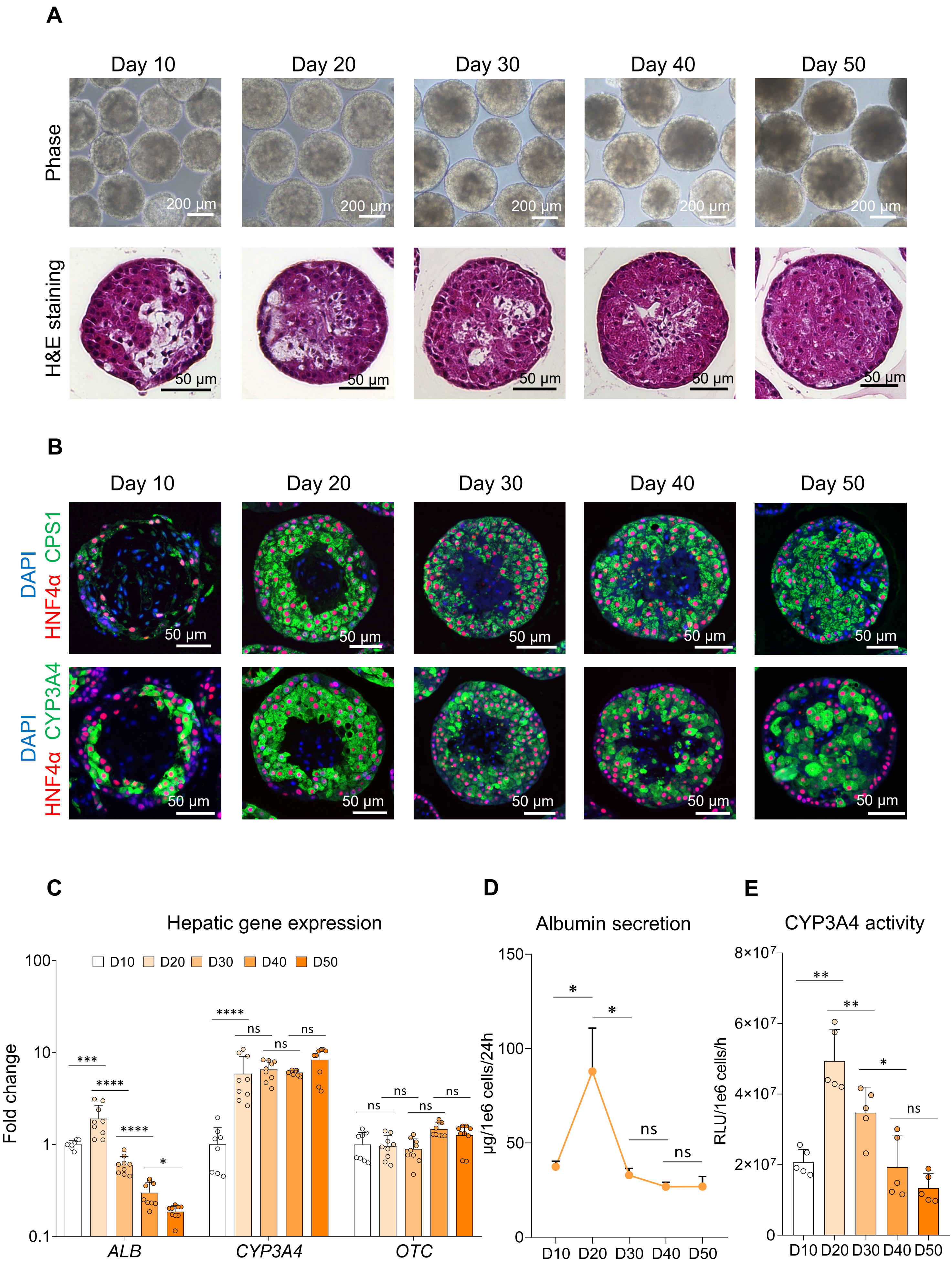

A
Day 10
Day 20
Day 30
Day 40
Day 50
Phase
H&E staining
200 µm
50 µm
B
DAPI HNF4α CPS1
DAPI HNF4α CYP3A4
50 µm
C
Hepatic gene expression
D10
D20
D30
D40
D50
Fold change
100
10
1
0.1
ALB
CYP3A4
OTC
ns
D
Albumin secretion
µg/1e6 cells/24h
150
100
50
0
D10
D20
D30
D40
D50
E
CYP3A4 activity
RLU/1e6 cells/h
8×10⁷
6×10⁷
4×10⁷
2×10⁷
0

Figure S5: Viability and functionality of Spin-LO within 50 days of culture.

A Upper panel shows the morphology of Spin-LOs from day 10 to day 50. Lower panel shows the H&E staining of Spin-LOs sections. **B** Immunofluorescence co-staining of HNF4a and functional marker (CYP3A4 and CPS1) of Spin-LOs from day 10 to day 50. **C** Expression of hepatic functional genes (*ALB*, *CYP3A4*, *OTC*) from day 10 to day 50 (day 10: $n = 8$, day 20-50: $n = 9$). Fold change is normalized to day 10. **D** Albumin secretion in supernatant of Spin-LOs from day 10 to day 50 ($n = 4$). **e** CYP3A4 activity assay of Spin-LOs from day 10 to day 50 ($n = 5$). Fold change is normalized to day 10. All data are shown as mean ± S.D. Statistical analyses were performed using Mann-Whitney test. $*P < 0.05$, $**P < 0.01$, $***P < 0.001$, $****P < 0.0001$, ns: not significant.

A

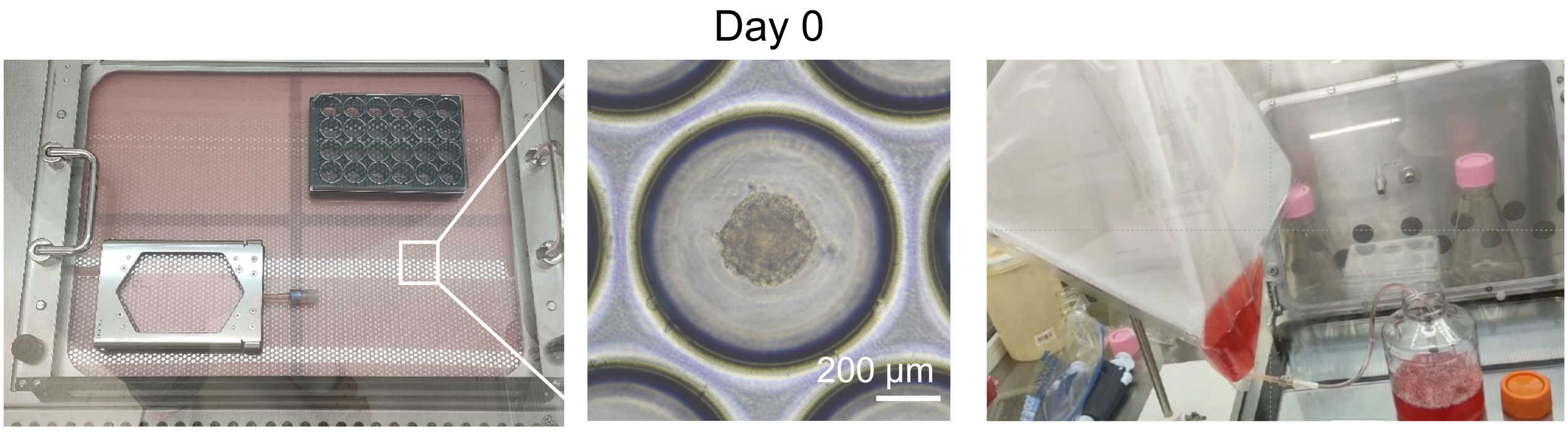

B

	Microwell plates	Microwell bags
Culture units required	~2000 plates	~30 bags
Collection time	~200 h	~5 h
Culture space required	High	Reduced
Labor required	High	Reduced
Manufacturing efficiency	Low	High

Figure S6: Scale-up generation of LOs with microwell bag.

A Left: Images showing a 24-well microwell plate, a small-sized microwell bag (50 cm^2), a large-sized microwell bag (1000 cm^2), and a callout of a single microwell from microwell bags. Right: Image showing LOs were harvested from the microwell bag. **B** Comparison between microwell plate and microwell bag for producing LOs for one patient treatment.

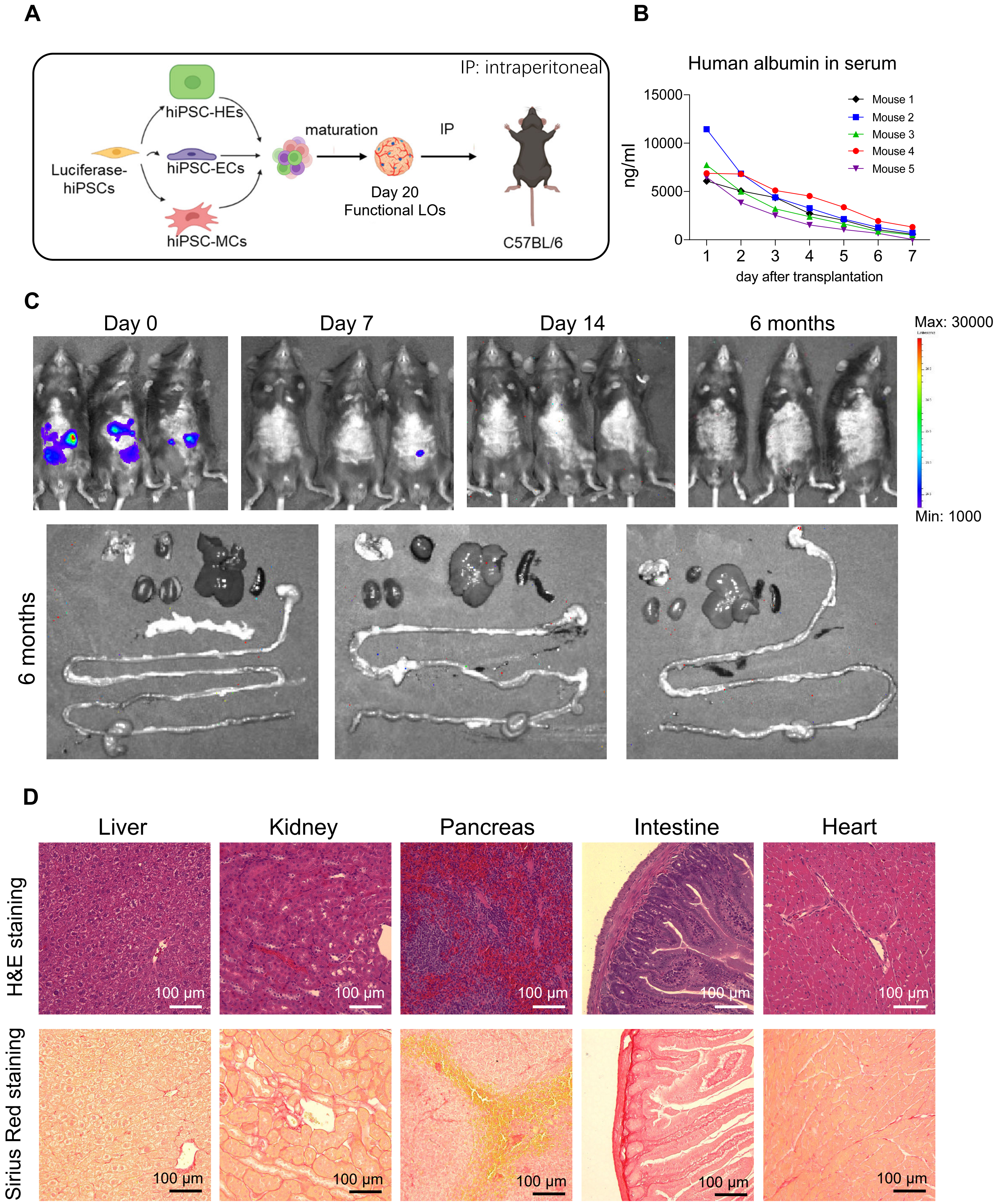

A
IP: intraperitoneal
hiPSC-HEs
Luciferase-hiPSCs
hiPSC-ECs
hiPSC-MCs
maturation
IP
Day 20
Functional LOs
C57BL/6
B
Human albumin in serum
ng/ml
15000
10000
5000
0
Mouse 1
Mouse 2
Mouse 3
Mouse 4
Mouse 5
1 2 3 4 5 6 7
day after transplantation
C
Day 0
Day 7
Day 14
6 months
Max: 30000
Min: 1000
6 months
D
Liver
Kidney
Pancreas
Intestine
Heart
H&E staining
Sirius Red staining
100 μm

Figure S7: Safety of intraperitoneal transplantation of functional LOs in immunocompetent mice.

A Schematic diagram showing the experimental design to transplant luciferase-labeled Bag-LOs into peritoneal cavity of C57BL/6 mice. IP: intraperitoneal. **B** Human albumin detected in sera of C57BL/6 mice during the first 7 days after transplantation (n = 5). **C** *In vivo* bioluminescence images of mice monitoring the viability of Bag-LOs after transplantation. **D** H&E staining and Sirius Red staining of several organs of recipient mice 6 months after LO transplantation.

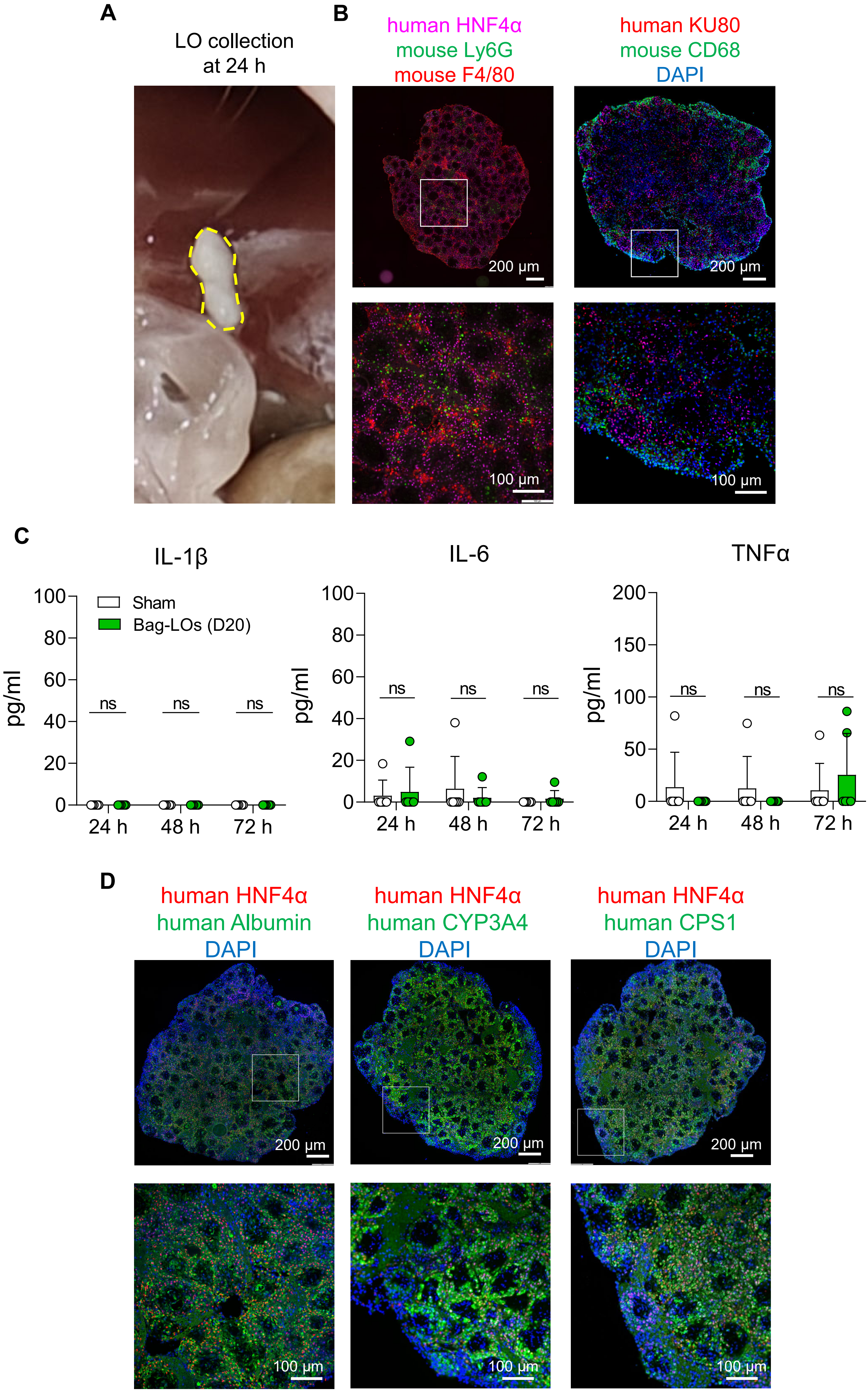
A
LO collection
at 24 h
B
human HNF4α
mouse Ly6G
mouse F4/80
human KU80
mouse CD68
DAPI
200 µm
100 µm
C
IL-1β
IL-6
TNFα
Sham
Bag-LOs (D20)
pg/ml
ns
24 h
48 h
72 h
D
human HNF4α
human Albumin
DAPI
human HNF4α
human CYP3A4
DAPI
human HNF4α
human CPS1
DAPI
200 µm
100 µm

Figure S8: Innate immune cell infiltration and hepatic function of transplanted liver organoids.

A LOs in peritoneal cavity 24 h post transplantation. Yellow arrow indicates the LO aggregation. **B** Immunofluorescence of mouse CD68, F4/80 and Ly6G with human KU80 and HNF4α of LO aggregates. **C** Inflammation factor (IL-1β, IL6, TNFα) detection in recipient mice sera after LO transplantation (n = 6). **D** Immunofluorescence of human HNF4α with Albumin, CYP3A4, and CPS1 of LO aggregates.

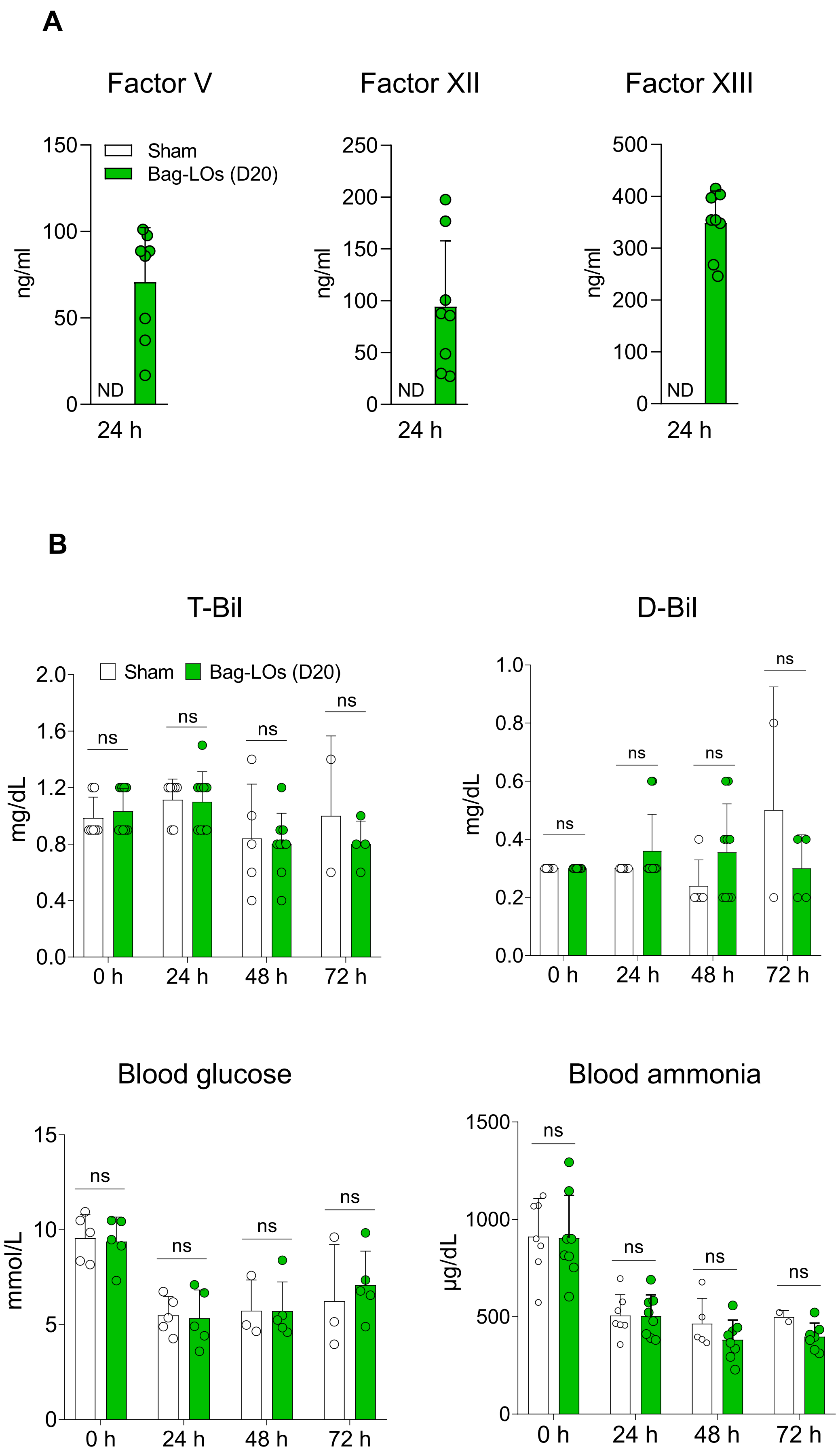

A
Factor V
Factor XII
Factor XIII
Sham
Bag-LOs (D20)
ng/ml
150
100
50
0
250
200
500
400
300
ND
24 h
B
T-Bil
D-Bil
Blood glucose
Blood ammonia
mg/dL
mmol/L
μg/dL
2.0
1.6
1.2
0.8
0.4
0.0
1.0
0.6
0.2
15
10
5
1500
1000
ns
0 h
24 h
48 h
72 h

Figure S9: Supplementary serum biochemical parameters measurement of mice after transplantation.

A Coagulation factor (V, XII, XIII) detection in sera of recipient mice after LO transplantation (*n* = 7) ND; Not detectable. **B** Serum biochemical parameters measurement (T-Bil, D-Bil, blood ammonia, blood glucose) of recipient mice after transplantation (*n* = 7).

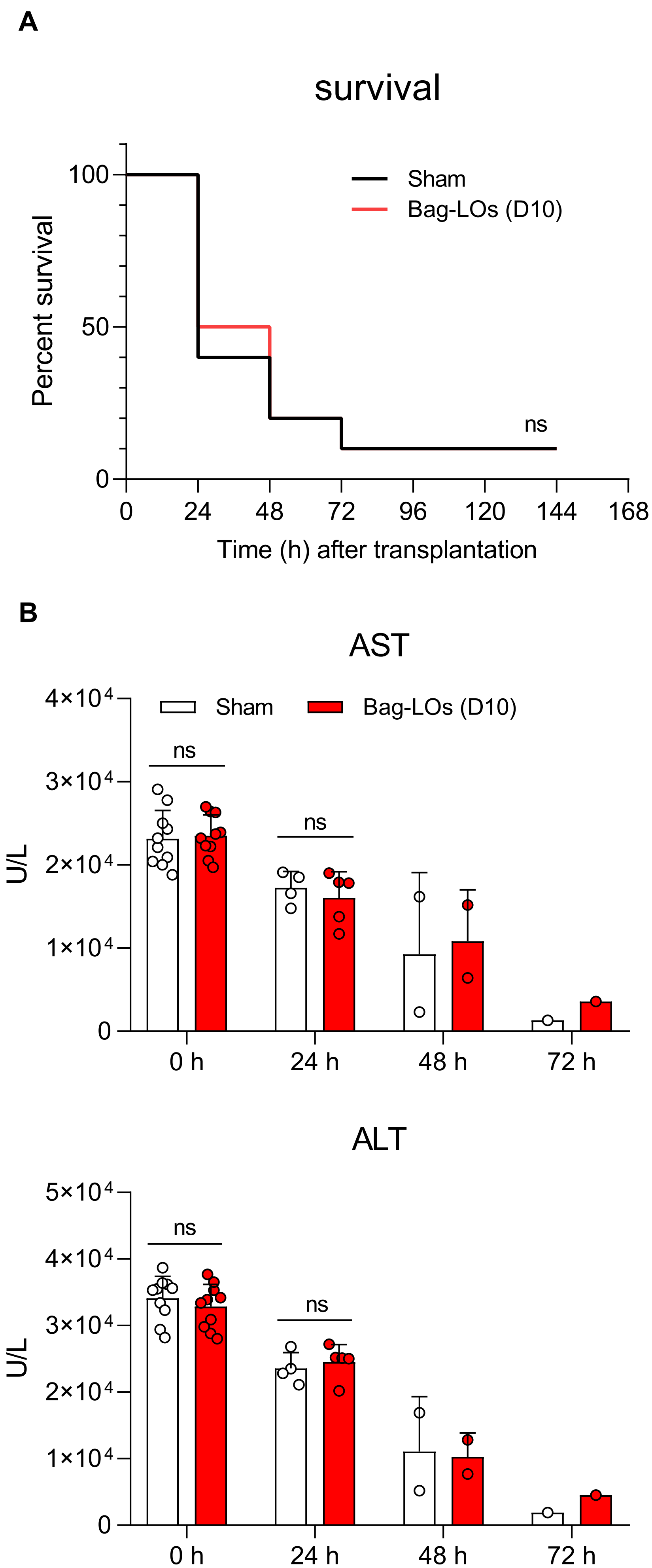
A
survival
Percent survival
100
50
0
Sham
Bag-LOs (D10)
ns
0
24
48
72
96
120
144
168
Time (h) after transplantation
B
AST
U/L
4×10⁴
3×10⁴
2×10⁴
1×10⁴
0
Sham
Bag-LOs (D10)
ns
ns
0 h
24 h
48 h
72 h
ALT
U/L
5×10⁴
4×10⁴
3×10⁴
2×10⁴
1×10⁴
0
ns
ns
0 h
24 h
48 h
72 h

Figure S10: Intraperitoneal transplantation of day 10 Bag-LOs into APAP-induced ALF mice.

A Kaplan-Meier survival curves of ALF mice transplanted with saline and day 10 Bag-LOs containing 3×10^6 HEs (*n* = 10). **B** Serum biochemical parameters measurements (AST, ALT) of mice after transplantation (*n* = 10).

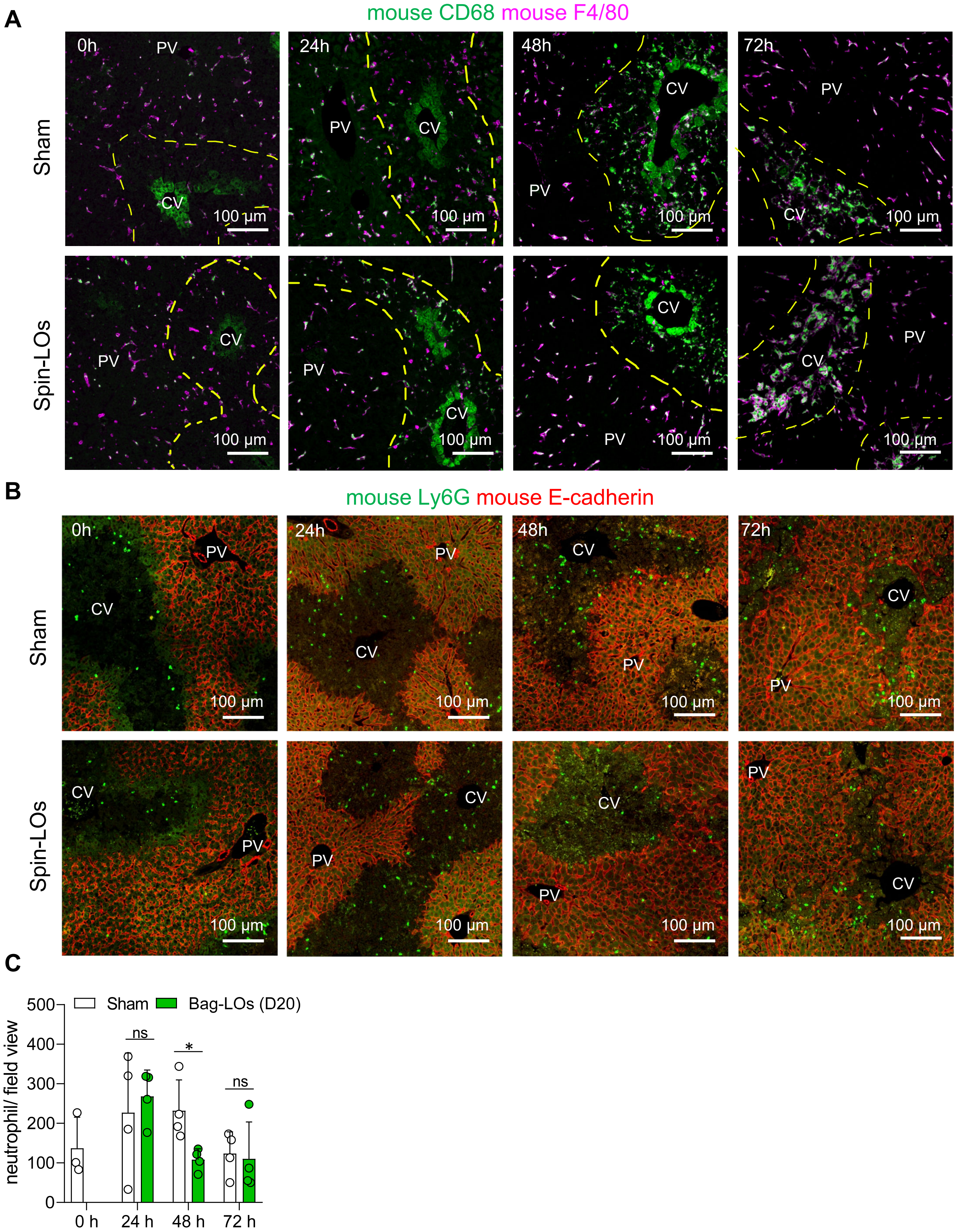
A
mouse CD68 mouse F4/80
Sham
Spin-LOs
0h
24h
48h
72h
PV
CV
100 µm
B
mouse Ly6G mouse E-cadherin
Sham
Spin-LOs
0h
24h
48h
72h
PV
CV
100 µm
C
Sham
Bag-LOs (D20)
neutrophil/ field view
500
400
300
200
100
0
ns
*
ns
0 h
24 h
48 h
72 h

Figure S11: Intraperitoneally transplanted day 20 Bag-LOs regulated liver regeneration and immune microenvironment in ALF mice.

A Immunofluorescence co-staining of mouse CD68 and F4/80 of liver of the mice. Yellow dotted line indicates the necrotic area. **B** Immunofluorescence co-staining of mouse Ly6G and E-cadherin of mice liver after transplantation. **C** Quantification analysis of mouse $Ly6G^{+}$ ratio in necrotic area (0h: n = 3, 24-72h: n = 4). All data are shown as mean ± S.D. Statistical analyses were performed using Mann-Whitney test. *$P < 0.05$, ns: not significant.

Tables

Table S1

Table S1: List of qPCR primers for human genes		
**Gene**	**Forward primer**	**Reverse primer**
ALB	AATGTTGCCAAGCTGCTGA	CTTCCCTTCATCCCGAAGTT
OTC	ACCTTCAGGCAGCTACTCCA	TTTGTCAGCAACAGCTTGGT
CPS1	CAAGTTTTGCAGTGGAATCG	ACTGGGTAGCCAATGGTGTC
AFP	TCCTTGTAAGTGGCTTCTTGAAC	TGTACTGCAGAGATAAGTTTAGCTGAC
G6PC	CCCTGTAACCTGTGAGACTGG	AAAGAGTAGATGTGACCATCACGTA
CYP3A5	ACGGTCATTGCTGTCTCCAACC	GTGACAGGCTTGCCTTTCTCTG
CYP3A4	GATGGCTCTCATCCCAGA CTT	AGTCCATGTGAATGGGTTCC
CYP2C9	TGGATGAAGGTGGCAATTTT	AGGAATAAAAACAGCTCCATGC
CYP7A1	GCTTATTCTTGGAATTAGGAGAAGG	TTGGCACCAAATTGCAGAG
CYP2C19	ACTATTGAAAACTTGGTAATCACTGC	TCTCAGGGTTGTGCTTGTTG
CYP2E1	GAGCACCATCAATCTCTGGACC	CACGGTGATACCGTCCATTGTG
CYP2B6	ACAGTGTGGAGAAGCACCGTGA	GGTTGAGGTTCTGGTGGCTGAA
MT-ND1	ATGGCCAACCTCCTACTCCTCATT	TTATGGCGTCAGCGAAGGGTTGTA
MT-ND2	CTAGCCCCCATCTCAATCATA	GAATGCGGTAGTAGTTAGGAT
MT-ND3	CCCTACCATGAGCCCTACAAACAA	AGTCACTCATAGGCCAGACTTAGG
MT-ND4	ACAAGCTCCATCTGCCTACGACAA	TTATGAGAATGACTGCGCCGGTGA
MT-ND5	ATCGGTTTCATCCTCGCCTTAGCA	ACCTAATTGGGCTGATTTGCCTGC
MT-ND6	AGGATTGGTGCTGTGGGTGAAAGA	ATAGGATCCTCCCGAATCAACCCT
MT-CO1	ACCCTAGACCAAACCTACGCCAAA	TAGGCCGAGAAAGTGTTGTGGGAA
ACTB	CCCCGCGAGCACAGC	CCACGATGGAGGGGAAGAC

Table S2

Table S2: List of antibodies for immunofluorescence staining			
**Primary Antibodies**	**Sources**	**Catalog**	**Dilution**
Polyclonal Goat Anti-Human ALB	Bethyl	Cat#A80-129A	1:100
Polyclonal Rabbit Anti-Human A1AT	Agilent	Cat#GA500	1:100
Monoclonal Mouse Anti-HNF4a	Perseus Proteomics	Cat# PP-H1415-0C	1:100
Polyclonal Rabbit Anti-Ki67	Abcam	Cat# ab15580	1:100
Monoclonal Mouse Anti-Human E-cadherin	BD Biosciences	Cat# 610182	1:100
Monoclonal Rabbit Anti-Human CYP3A4	Abcam	Cat# ab124921	1:100
Polyclonal Rabbit Anti-Human CPS1	Abcam	Cat# ab45956	1:100
Monoclonal Mouse Anti-Human Mitochondria	Abcam	Cat#ab92824	1:100
Monoclonal Mouse Anti-Human ZO1	Invitrogen	Cat#33-9100	1:100
Monoclonal Rabbit Anti-Mouse CD68	Abcam	Cat#ab283654	1:100
Monoclonal Rabbit Anti-Mouse F4/80	Cell Signaling Technology	Cat#D2S9R	1:100
Monoclonal Rabbit Anti-Human Ku80	Cell Signaling Technology	Cat#C48E7	1:100
Monoclonal Rabbit Anti-Mouse Ly6G	Cell Signaling Technology	Cat#E6Z1T	1:100
**Secondary Antibodies**			
Donkey Anti-Rabbit IgG (H+L) Alexa Fluor 488	Invitrogen	Cat#A-21206	1:500
Donkey Anti-Rabbit IgG (H+L) Alexa Fluor 647	Invitrogen	Cat#A-31573	1:500
Donkey Anti-Goat IgG (H+L) Alexa Fluor 555	Invitrogen	Cat#A-21432	1:500
